# Impact of Chemotherapy on Implant-Based Breast Reconstruction in Breast Cancer Patients: A Nationwide, Retrospective, Cohort Study

**DOI:** 10.3390/cancers17122053

**Published:** 2025-06-19

**Authors:** Jin Ah Lee, Hye Sun Lee, Soyoung Jeon, Dooreh Kim, Young Joo Lee, Soo Youn Bae, Woo-Chan Park, Chang Ik Yoon, Jangyoun Choi

**Affiliations:** 1 Division of Breast Surgery, Department of Surgery, Seoul St. Mary’s Hospital, College of Medicine, The Catholic University of Korea, Seoul 06591, Republic of Korea; 2 Biostatistics Collaboration Unit, Yonsei University College of Medicine, Seoul 03722, Republic of Korea; 3 Department of Plastic and Reconstructive Surgery, Seoul St. Mary’s Hospital, College of Medicine, The Catholic University of Korea, Seoul 06591, Republic of Korea

**Keywords:** breast cancer, implant-based breast reconstruction, capsular contracture, chemotherapy

## Abstract

Background: Implant-based breast reconstruction (IBBR) is a widely adopted technique following mastectomy in breast cancer patients. However, the impact of chemotherapy type and duration on the development of capsular contracture remains unclear. Methods: This nationwide, retrospective, cohort study used Health Insurance Review and Assessment Service data to identify breast cancer patients who received chemotherapy and underwent immediate IBBR between January 2015 and December 2018. Follow-up continued until January 2024, with a median follow-up of 5.2 years. A total of 4303 patients (direct-to-implant [DTI], n = 2083; tissue expander insertion [TEI], n = 2220) were included. Results: Chemotherapy type and duration were not significantly associated with capsular contracture risk in either the DTI or TEI groups. In the DTI cohort, no significant difference in contracture incidence was found between neoadjuvant and adjuvant chemotherapy before or after matching (*p* = 0.056 and *p* = 0.121, respectively). In the TEI cohort, an initially significant difference (*p* = 0.019) was no longer observed after matching (*p* = 0.213). Similarly, chemotherapy duration (≤12 weeks vs. >12 weeks) did not impact contracture risk in either cohort. Multivariate analysis identified age, radiotherapy, lymphedema, and axillary lymph node dissection (ALND) as independent risk factors for contracture (*p* < 0.005). Comorbidities, such as diabetes and autoimmune diseases, also showed no significant association with contracture risk. Conclusions: These findings suggest that chemotherapy decisions should not be guided by contracture concerns. Instead, optimizing reconstruction outcomes should focus on modifiable factors, such as radiotherapy, lymphedema, and ALND.

## Simple Summary

Breast reconstruction using implants is commonly performed after mastectomy in breast cancer patients. Some patients worry that chemotherapy might increase the risk of complications, such as capsular contracture, a condition where scar tissue tightens around the implant. To address this concern, we studied over 4000 Korean breast cancer patients who had implant-based reconstruction and received chemotherapy. We found that neither the type nor duration of chemotherapy increased the risk of capsular contracture. However, other factors, like radiation therapy, lymphedema, and extensive lymph node surgery, were linked to a higher risk. These results suggest that chemotherapy should not be avoided due to fears about contracture. Instead, reconstruction should focus on improving outcomes and reducing other risks, such as managing lymphedema and carefully planning radiation and lymph node surgery.

## 1. Introduction

Breast construction is essential for enhancing the quality of life and psychological well-being of breast cancer patients undergoing total mastectomy. As a key aspect of postmastectomy care, it has been shown to improve body image, self-esteem, and overall patient satisfaction [1]. Advances in surgical techniques have resulted in two primary reconstruction methods: autologous reconstruction and implant-based breast reconstruction (IBBR). IBBR remains widely performed and is a preferred option for many patients and surgeons [2,3]. It offers benefits such as shorter operative times, quicker recovery, and the avoidance of donor-site morbidity associated with autologous tissue harvesting. However, IBBR also has drawbacks, including risks of capsular contracture, implant rupture, infection, and less natural esthetic outcomes compared to autologous reconstruction, along with potential long-term implant-related complications [4].

Neoadjuvant and adjuvant chemotherapy are essential in breast cancer treatment, improving survival outcomes and lowering recurrence risks [5]. However, these systemic therapies may impact breast reconstruction success by affecting tissue healing, vascularization, and immune response. Chemotherapy-induced cytotoxicity can impair fibroblast function, delay wound healing, and hinder neovascularization, all of which are crucial for optimal reconstruction outcomes [6]. In particular, different chemotherapeutic agents have distinct biological effects. Taxanes (e.g., paclitaxel, docetaxel) have been shown to inhibit angiogenesis by suppressing endothelial cell proliferation and migration, thereby impairing neovascularization and delaying tissue repair [7]. Anthracyclines, such as doxorubicin, may impair wound healing by inducing oxidative stress and chronic inflammation through free radical generation, thereby disrupting normal tissue repair processes [8]. While previous studies have examined chemotherapy’s effect on reconstruction failure rates, the findings remain inconsistent. Some suggest a significant increase in complications, while others report no substantial impact [9,10,11,12]. Moreover, many studies are limited by short follow-up periods and single-institution data.

This study aimed to assess the risk factors and incidence of breast reconstruction failure in chemotherapy-treated breast cancer patients using nationwide cohort data.

## 2. Materials and Methods

This study was conducted as a retrospective cohort analysis using data from the Health Insurance Review and Assessment Service (HIRA) of South Korea. The HIRA database includes comprehensive medical claims information, such as patient demographics, disease registration dates, diagnostic codes, procedure codes, and prescription records. Due to this study’s retrospective design, written consent was not required. All data were anonymized and managed in compliance with the Health Insurance Portability and Accountability Act of Korea. The study protocol was approved by the Institutional Review Board (IRB) of the Catholic University of Korea (local IRB number: KC23RISI0200).

### 2.1. Study Design and Patient Enrollment Criteria

This nationwide, population-based cohort study identified patients diagnosed with ductal carcinoma in situ (DCIS, International Classification of Disease [ICD]-10 code D05) or invasive breast cancer (ICD-10 code C50) between January 2015 and December 2018. From this group, individuals who underwent total mastectomy within 1 year of diagnosis were selected, with the corresponding procedure codes provided in Table S1. This study specifically included breast cancer patients who received chemotherapy and underwent immediate breast reconstruction using either direct-to-implant (DTI) or two-stage tissue expander insertion (TEI) techniques (procedure codes: N7148, N7149).

To ensure accurate classification, only patients with primary DCIS or breast cancer who underwent curative surgery within 1 year of diagnosis were included. Those diagnosed with another malignancy (ICD-10 code: any C codes) within 2 years before or 6 months after curative surgery were excluded (Figure S1). Patients who underwent bilateral mastectomy were also excluded, as our analysis was conducted on a per-patient basis. Including both breasts from a single individual could lead to an overrepresentation of complications and violate the assumption of independent observations, potentially introducing analytical bias. Moreover, the extent of surgical intervention and systemic inflammatory response may differ in bilateral cases. Additionally, we excluded patients with a history of prior breast augmentation or mammoplasty, including those who underwent capsulectomy due to prior augmentation, as these individuals may have altered tissue characteristics or residual implant-related effects that could confound the assessment of postoperative complications, such as capsular contracture or tissue compatibility. Among the eligible patients, those who underwent capsulectomy (procedure code: N7151) were identified for further analysis, while individuals who only had implant replacement surgery were excluded, as implant changes may have been due to patient preference rather than medical necessity. This study defined the endpoints differently for the DTI and TEI cohorts: in the TEI cohort, the endpoint was capsulectomy with implant exchange, whereas in the DTI cohort, the endpoint was capsulectomy regardless of implant replacement.

Chemotherapy status was determined by identifying patients who received at least one chemotherapy session from 1 year before the enrollment day to the last recorded prescription. Those who underwent chemotherapy within 1 year before the enrollment day were classified as receiving neoadjuvant chemotherapy, while those who started chemotherapy after the enrollment day were categorized as receiving adjuvant chemotherapy, regardless of the agents used. Chemotherapy duration was further analyzed by dividing the patients into two groups based on a threshold of four cycles or 12 weeks. In Korea, human epidermal growth factor receptor 2 (HER2)-targeted therapy, such as trastuzumab, is only administered in combination with cytotoxic chemotherapy due to reimbursement regulations. Therefore, patients who received HER2-targeted therapy were included in the chemotherapy group, and HER2-targeted agents were not categorized separately.

To assess the risk factors for capsular contracture, prescription records were reviewed to identify chemotherapy, endocrine therapy, and targeted therapy using drug prescription codes (Table S2). Diagnostic codes were also analyzed to determine the presence of comorbidities, including diabetes, dyslipidemia, and autoimmune diseases (Table S3). Autoimmune diseases included rheumatoid arthritis, lupus erythematosus, systemic sclerosis, Sicca syndrome, Behcet’s disease, autoimmune thyroiditis, atopic dermatitis, vitiligo, and psoriasis, while autoimmune hepatitis and adrenalitis were excluded due to the absence of cases in this cohort.

To evaluate comorbidity burden, the Charlson Comorbidity index (CCI), a validated tool for estimating 10-year survival in patients with multiple chronic conditions, was used. The CCI score helped predict complication incidence in patients with multiple comorbidities, with analyses conducted using the weighted index (Table S4) [13,14,15].

### 2.2. Study Outcomes

The primary endpoint was to evaluate the incidence of capsular contracture and identify associated risk factors in breast cancer patients undergoing reconstruction, categorized by chemotherapy type (neoadjuvant vs. adjuvant) in both DTI and TEI reconstruction. The secondary endpoint was to assess the incidence and risk factors for capsular contracture based on chemotherapy duration within the same reconstruction subgroups, regardless of chemotherapy type.

### 2.3. Statistical Analysis

Baseline demographic and clinical characteristics were compared using *t*-tests for continuous variables and chi-squared tests for categorical variables. The cumulative incidence of capsular contracture was illustrated with Kaplan–Meier curves and compared using log-rank tests. Risk factors were identified using Cox proportional hazard models to estimate hazard ratios and 95% confidence intervals, adjusting for potential confounders. The multivariable Cox proportional hazard model was applied using the Enter method. To minimize baseline differences between treatment groups, 1:1 propensity score matching was performed using a logistic regression model. The covariates included in the propensity score model were age, endocrine therapy, HER2-targeted therapy, radiotherapy, lymphedema, diagnosis code, axillary surgery, diabetes, dyslipidemia, autoimmune disease, steroid medication, and CCI. Statistical significance was defined as a two-tailed *p*-value of <0.05. All randomization procedures and statistical analyses were conducted using SAS software (version 9.4, SAS Institute Inc., Cary, NC, USA).

## 3. Results

### 3.1. Patient Flow Diagram of the Cohort

**Figure 1:**
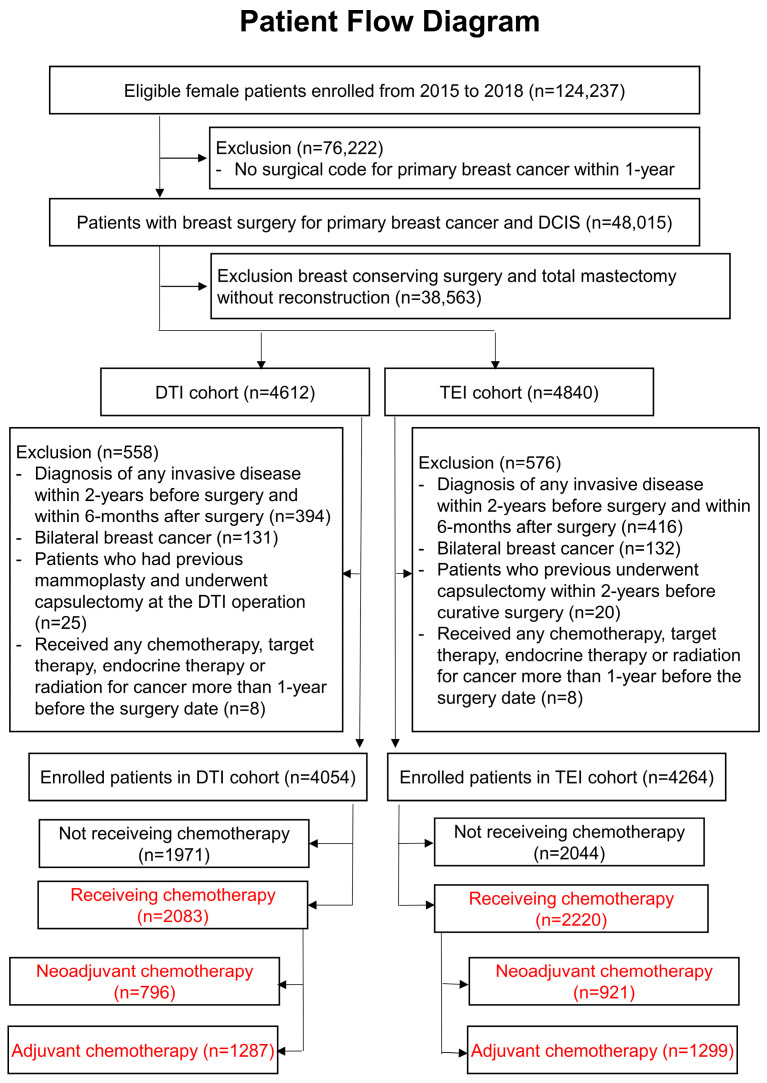
Flowchart of patient selection in the retrospective cohort study.

Between 2015 and 2018, 124,237 patients were diagnosed with DCIS or invasive breast cancer (Figure 1). Of these, 76,222 patients who did not undergo surgery within 1 year of diagnosis were excluded, leaving 48,015 patients who underwent surgery. Among them, 38,563 patients who did not undergo breast reconstruction were further excluded. As a result, 4612 patients underwent DTI reconstruction, while 4840 underwent TEI reconstruction.

Between 2015 and 2018, 124,237 patients diagnosed with breast cancer or DCIS were identified from the HIRA database. Of these, 76,222 patients who did not undergo curative surgery within 1 year were excluded, leaving 48,015 patients. Further exclusions based on specific criteria resulted in a final cohort of 8318 patients, including 4054 in the DTI cohort and 4264 in the TEI cohort. After removing patients who did not receive chemotherapy, 2083 patients in the DTI cohort and 2220 patients in the TEI cohort were analyzed. Propensity score matching was conducted at a 1:1 ratio.

After excluding patients with another invasive malignancy within 2 years before or 6 months after surgery, those with bilateral breast cancer, a history of prior capsulectomy or mammoplasty, or those who had received chemotherapy, target therapy, or radiotherapy within 1 year before surgery, the final cohort included 2083 patients in the DTI group and 2220 in the TEI group who had received chemotherapy.

### 3.2. Demographics and Incidence by Chemotherapy Type

#### 3.2.1. DTI Cohort

Table 1 compares the clinical characteristics of patients who underwent DTI reconstruction, categorized by chemotherapy type. Before matching, among the 2083 patients in the DTI cohort, 796 received neoadjuvant chemotherapy, while 1287 received adjuvant chemotherapy. The incidence of capsulectomy was 11.3% in the neoadjuvant group and 9.0% in the adjuvant group, with no statistically significant difference between them. Significant differences were observed between the groups in terms of age (*p* < 0.001), CCI (*p* < 0.001), and breast cancer treatment modalities, including endocrine therapy, chemotherapy, and radiation therapy. Additionally, the incidence of lymphedema (*p* = 0.005) and the type of axillary surgery performed (*p* < 0.001) differed significantly. However, no significant differences were found in comorbidities such as diabetes mellitus (DM) and autoimmune diseases. Steroid use was also not significantly associated with capsular contracture (*p* = 0.185). After matching, no significant differences remained across all variables between the two groups.

**Figure 2:**
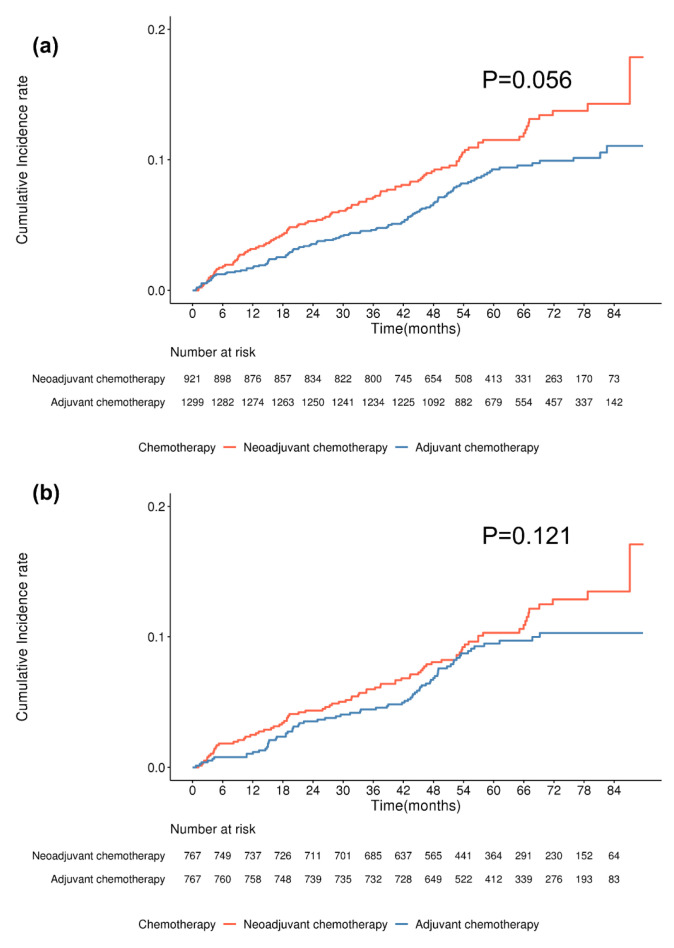
Cumulative incidence of capsular contracture in breast cancer patients undergoing DTI reconstruction by chemotherapy type. (**a**) Before matching, the cumulative incidence of capsular contracture did not significantly differ between chemotherapy types (*p* = 0.056, log-rank test). (**b**) After matching, the difference remained statistically nonsignificant (*p* = 0.121, log-rank test).

The median follow-up duration for this study was 61.92 ± 17.90 months. Figure 2 illustrates the cumulative incidence of capsular contracture in patients who underwent DTI reconstruction, categorized by chemotherapy type. The incidence was slightly higher in the neoadjuvant chemotherapy group compared to the adjuvant chemotherapy group, but the difference was not statistically significant (Figure 2a, *p* = 0.056). After matching, the differences between the groups were further reduced, with no statistically significant association observed (Figure 2b, *p* = 0.121).

#### 3.2.2. TEI Cohort

Table 2 summarizes the clinical characteristics of patients who underwent TEI reconstruction, grouped by chemotherapy type. Among the 2220 patients, 921 received neoadjuvant chemotherapy, while 1299 received adjuvant chemotherapy. The incidence of capsulectomy was 11.3% in the neoadjuvant group and 9.1% in the adjuvant group, with no statistically significant difference (*p* = 0.088). Significant differences were found between the two groups in terms of age, CCI, and breast cancer treatment modalities, including endocrine therapy, chemotherapy, and radiation therapy. Additionally, the incidence of lymphedema (*p* = 0.046) and the type of axillary surgery performed (*p* < 0.001) varied significantly. However, no significant differences were noted in comorbidities such as DM and autoimmune diseases. Likewise, steroid use was not significantly associated with capsular contracture incidence (*p* = 0.059). After matching, no significant differences remained across all variables.

**Figure 3:**
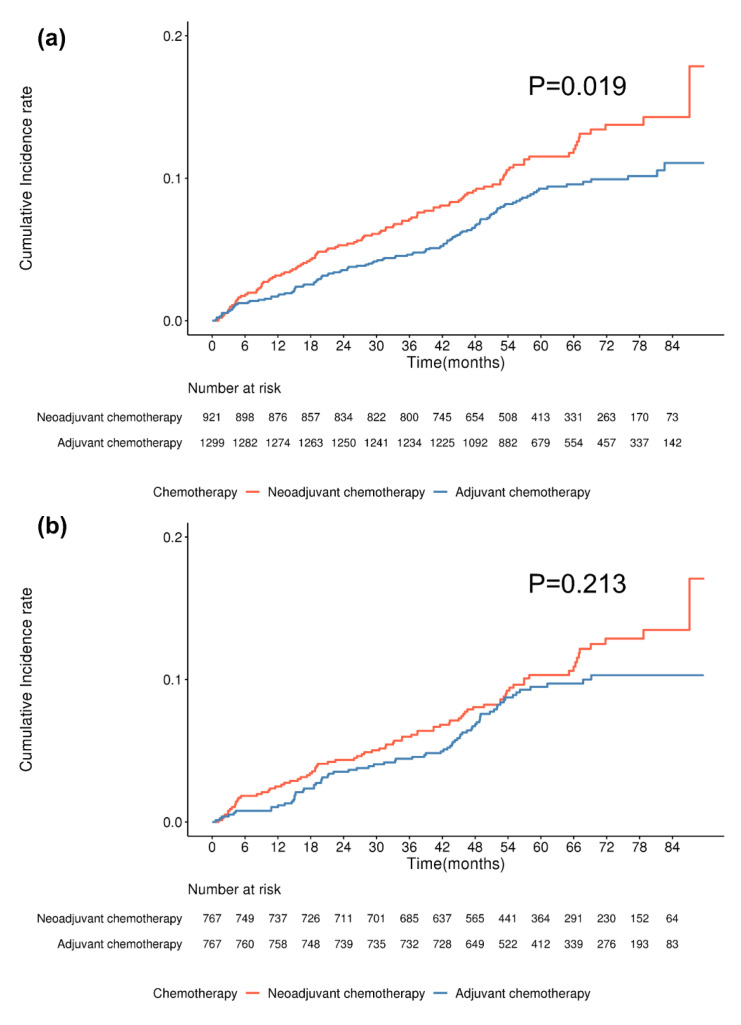
Cumulative incidence of capsular contracture in breast cancer patients undergoing TEI by chemotherapy type. (**a**) Before matching, a significant difference was observed in the cumulative incidence of capsular contracture between chemotherapy types (*p* = 0.019, log-rank test). (**b**) After matching, this difference was no longer statistically significant (*p* = 0.213, log-rank test).

The median follow-up period for this study was 60.17 ± 19.30 months. Figure 3 illustrates the cumulative incidence of capsular contracture in patients who underwent TEI reconstruction, categorized by chemotherapy type. Initially, the incidence was higher in the neoadjuvant chemotherapy group, showing a statistically significant difference (Figure 3a, *p* = 0.019). However, after matching, this difference was no longer significant (Figure 3b, *p* = 0.213).

### 3.3. Risk Factors for Capsular Contracture by Chemotherapy Type

#### 3.3.1. DTI Cohort

The risk factors for capsular contracture in breast cancer patients undergoing DTI reconstruction were assessed using Cox proportional hazard models (Table 3). In the univariate analysis, age, CCI, radiotherapy, and lymphedema were identified as significant risk factors. Multivariate analysis was performed using two models:

Model 1 included autoimmune diseases (rheumatoid arthritis, lupus erythematosus, or Behcet’s disease) as a single composite variable.

Model 2 analyzed each autoimmune disease separately, providing a fully adjusted model to control for potential confounders.

Both models consistently identified age, radiotherapy, lymphedema, and the type of axillary surgery as significant risk factors for capsular contracture.

#### 3.3.2. TEI Cohort

Risk factors for capsular contracture in TEI reconstruction patients were also analyzed using Cox proportional hazard models (Table 4). In the univariate analysis, age, chemotherapy type, radiotherapy, lymphedema, and the axillary surgery method were identified as significant risk factors. These variables remained significant in both Model 1 and Model 2 after multivariate analysis.

### 3.4. Demographics and Incidence of Contracture Based on Chemotherapy Duration

Additional analyses were performed to evaluate the effect of chemotherapy duration in both cohorts (Tables S5 and S6). Patients were grouped based on chemotherapy duration into those who received chemotherapy for up to four cycles (≤12 weeks, n = 1047) and those who underwent more than five cycles (>12 weeks, n = 1173). In both the DTI and TEI cohorts, significant differences were observed in CCI, radiotherapy, lymphedema, and axillary surgery between these groups. Moreover, the prevalence of autoimmune thyroiditis was significantly higher in patients who received more than five cycles of chemotherapy (*p* = 0.037).

The cumulative incidence of capsular contracture based on chemotherapy duration was analyzed in both cohorts (Figures S2 and S3). No statistically significant differences were observed between patients who received chemotherapy for up to four cycles (≤12 weeks) and those who underwent more than five cycles (>12 weeks).

### 3.5. Risk Factors for Capsular Contracture Based on Chemotherapy Duration

Risk factors for capsular contracture were evaluated according to chemotherapy duration in both the DTI and TEI cohorts (Tables S7 and S8). In the DTI cohort, age, radiotherapy, and lymphedema were consistently identified as significant risk factors across all models. Similarly, in the TEI cohort, age, radiotherapy, and lymphedema remained significant in the univariate analysis. However, unlike the DTI cohort, axillary surgery was a significant risk factor in all models for the TEI cohort. Notably, after matching, atopic dermatitis was identified as a significant risk factor for capsular contracture in both cohorts.

## 4. Discussion

This study indicates that neither the timing nor the duration of chemotherapy significantly affects the risk of capsular contracture following IBBR. Instead, key risk factors for capsular contracture include patient age, radiotherapy, lymphedema, and the type of axillary surgery performed. These findings suggest that reconstructive decisions should not be constrained by chemotherapy timing, allowing for greater flexibility in planning without concerns about chemotherapy-induced contracture. Instead, greater attention should be paid to factors such as radiotherapy, lymphedema, and axillary surgery type when assessing the risk of implant-related complications. Surgeons should carefully evaluate these factors to enhance reconstruction outcomes and minimize postoperative complications. The HIRA claims database, which covers over 97% of the Korean population, provides a highly representative and comprehensive source of nationwide medical data. Capsular contracture after IBBR was identified using the exclusive procedure code N7151, a code that is strictly regulated and routinely audited with supporting medical records for reimbursement purposes, thereby ensuring high coding accuracy.

Variations in capsular contracture risk were observed between DTI and TEI reconstruction. In the TEI cohort, a statistically significant difference in contracture risk was found between the neoadjuvant and adjuvant chemotherapy groups (*p* = 0.019); however, this significance was lost after propensity score matching (*p* = 0.213). In contrast, no significant difference in contracture risk was observed in the DTI cohort based on chemotherapy type. These results suggest that TEI reconstruction may be more vulnerable to early treatment-related effects, possibly due to differences in initial tissue expansion and healing processes. Further research is needed to determine whether specific surgical or adjuvant therapy modifications could help reduce these risks.

The incidence of capsular contracture generally increased at a steady rate until approximately 60 months postoperatively. This pattern differs from previous studies, which reported a peak incidence around 1 year after surgery. This finding suggests that earlier studies with shorter follow-up periods may have missed this longer-term trend. Also, differences in medical access and long-term follow-up protocol within the national health insurance system may play a role in this pattern.

Previous studies have reported conflicting findings on the impact of chemotherapy on breast reconstruction outcomes [9,10,11,12,16,17,18,19,20,21,22,23,24]. Some studies have suggested that neoadjuvant or adjuvant chemotherapy increases the risk of complications, including tissue expander loss, wound healing issues, and higher postoperative complication rates, particularly in implant-based reconstruction. However, many of these studies have limitations that hinder the generalizability of their findings, including small sample sizes [10,16,21,23]; short-term follow-up durations [17,24]; restriction to specific reconstruction types, such as TEI or autologous tissue reconstruction [11,20,21]; and single-center designs [22]. Moreover, few studies have adequately distinguished between immediate versus delayed reconstruction or accounted for the differences between neoadjuvant and adjuvant chemotherapy. Our study addresses these limitations by utilizing a large, nationwide cohort with long-term follow-up data and a clear distinction between different reconstruction methods and chemotherapy timing. This comprehensive design enables a more robust assessment of the association between chemotherapy and reconstruction outcomes.

Consistent with the prospective study by Hart et al. [9], our study also found no significant link between neoadjuvant or adjuvant chemotherapy and postoperative complications in immediate breast reconstruction. However, while their study primarily assessed patient-reported outcomes, our study focused on clinically significant complications requiring surgical intervention, offering a more objective evaluation of postoperative morbidity. Unlike previous studies that combined DTI and TEI reconstruction into a single group, our study analyzed these two cohorts separately, allowing for a more precise assessment of surgical outcomes for each reconstruction method. Additionally, we conducted a detailed analysis based on chemotherapy duration, offering new insights into whether the length of chemotherapy exposure affects postoperative complications. Furthermore, while Hart et al.’s study had a median follow-up of approximately 2 years, our study followed patients for a longer period of 5.2 years. This extended follow-up provided a more comprehensive assessment of long-term surgical outcomes, enhancing the reliability of our findings. These results contribute to more informed decision-making for both patients and clinicians when planning breast reconstruction in the context of chemotherapy.

Interestingly, axillary lymph node dissection (ALND) was associated with a decreased risk of capsular contracture in our cohort. This finding may appear counterintuitive, as ALND is typically considered a more invasive surgical procedure. However, this result likely reflects real-world treatment patterns based on nodal burden. In Korea, patients with extensive nodal involvement (e.g., N2 or higher) are commonly treated with both ALND and radiotherapy, which may contribute to an increased risk of contracture. In contrast, for patients with limited nodal disease (e.g., N1), ALND may be performed without additional radiotherapy, whereas those undergoing only sentinel lymph node biopsy are more likely to receive regional radiotherapy based on the AMAROS trial [25]. This differential in radiation exposure may partly explain the observed protective association of ALND with capsular contracture.

In our study, multivariate analysis identified age, radiotherapy, lymphedema, and ALND as independent risk factors for capsular contracture (*p* < 0.005). The association observed between radiotherapy, lymphedema, and capsular contracture are supported by biologically plausible mechanisms. Radiotherapy causes vascular endothelial injury, leading to impaired microcirculation and local tissue hypoxia [26]. These conditions promote fibroblast activation and myofibroblast differentiation, which drive collagen overproduction and fibrotic capsule formation. Radiation exposure also upregulates profibrotic cytokines, particularly transforming growth factor-beta (TGF-ß), further contributing to excessive extracellular matrix deposition [27]. Lymphedema, often secondary to axillary surgery or radiotherapy, impairs lymphatic drainage and facilitates the accumulation of interstitial fluid and inflammatory mediators [28,29]. This chronic inflammatory environment disrupts normal wound healing and compromises local immune surveillance, promoting sustained fibroblast activity and abnormal scar formation. These mechanistic insights help contextualize our findings and underscore the clinical relevance of managing these risk factors in patients undergoing implant-based reconstruction. A major strength of this study is the inclusion of a large, nationwide cohort, which addresses the limitations of single-institution studies and improves the generalizability of our findings. Additionally, the long-term follow-up period enables a more accurate assessment of contracture risk over time. Another key strength is the consideration of chemotherapy duration, a factor often overlooked in previous research [9]. By examining both the chemotherapy sequence and duration of chemotherapy, this study offers a more comprehensive understanding of its impact on IBBR outcomes. However, this study has several limitations. First, its retrospective design may introduce residual confounding. Second, while the HIRA database provides a large and representative sample, it lacks detailed clinical information, such as surgical techniques, implant types, and patient-specific factors, like body mass index. Additionally, this study did not evaluate specific radiotherapy parameters, including dose, fractionation, and radiation field, in relation to contracture risk. Moreover, the reduction in sample size after propensity score matching may have limited the statistical power for detecting subgroup differences, raising the possibility of a type II error. The analysis also did not distinguish chemotherapy regimens, such as the inclusion of anthracycline, taxanes, or HER2-targeted agents, which may differentially affect surgical outcomes. Future analyses are needed to assess the impact of specific chemotherapy regimens and targeted therapies on the risk of implant-related complications. Lastly, capsular contracture was identified based on insurance claims data, which may exclude mild cases and preclude an analysis of contracture severity. Nonetheless, this approach ensures that the findings focus on clinically significant contracture requiring surgical intervention.

Although our results do not suggest an increased risk of capsular contracture in chemotherapy after IBBR, acute and subacute postoperative complications after IBBR can delay the initiation of adjuvant chemotherapy. A multidisciplinary collaboration between breast and plastic surgeons, with attention to mastectomy flap vascularity and strict infection prevention, is essential and support timely recovery. Such coordinated care is critical for minimizing adjuvant treatment delays and optimizing outcomes in patients undergoing IBBR. While this study offers important insights, further research is necessary to enhance our understanding of capsular contracture risk. Our findings should also be validated using large-scale datasets that include detailed surgical variables, implant types, racial diversity, and patient-reported outcomes. Further studies should incorporate broader definitions of contracture, such as Baker grading [30] and patient satisfaction scores, to account for milder cases that were not captured in this study.

## 5. Conclusions

The type and duration of chemotherapy were not significantly linked to capsular contracture after IBBR. Therefore, chemotherapy regimens should not be altered due to concerns about reconstruction outcomes. Future large-scale clinical studies are needed to explore factors influencing capsular contracture and to develop strategies for improving breast reconstruction outcomes in breast cancer patients.

## Figures and Tables

**Table 1:** Comparison of clinical characteristics of breast cancer patients undergoing DTI reconstruction after total mastectomy according to chemotherapy type.

	Before Matching			After Matching		
	Patients Receiving Neoadjuvant Chemotherapy, n = 796 (%)	Patients Receiving Adjuvant Chemotherapy, n = 1287 (%)	*p* Value	Patients Receiving Neoadjuvant Chemotherapy, n = 718 (%)	Patients Receiving Adjuvant Chemotherapy, n = 718 (%)	*p* Value
Capsulectomy only			0.088			0.192
Not performed	706 (88.7)	1171 (91.0)		637 (88.7)	652 (90.8)	
Performed	90 (11.3)	116 (9.0)		81 (11.3)	66 (9.2)	
Age (year)			<0.001			0.976
20–29	22 (2.8)	18 (1.4)		12 (1.7)	13 (1.8)	
30–39	184 (23.1)	217 (16.9)		148 (20.6)	140 (19.5)	
40–49	331 (41.6)	591 (45.9)		315 (43.9)	316 (44.0)	
50–59	216 (27.1)	358 (27.8)		200 (27.9)	209 (29.1)	
60–69	40 (5.0)	96 (7.5)		40 (5.6)	36 (5.0)	
70–79	3 (0.4)	7 (0.5)		3 (0.4)	4 (0.6)	
CCI (Weight number, mean ± SD)	4.14 ± 2.61	3.67 ± 2.27	<0.001	3.90 ± 2.44	3.85 ± 2.46	0.683
Endocrine therapy			0.005			0.465
Not performed	226 (28.4)	294 (22.8)		186 (25.9)	172 (24.2)	
Performed	570 (71.6)	993 (77.2)		532 (74.1)	544 (75.8)	
HER2-target therapy			0.570			0.689
Not performed	541 (68.0)	890 (69.2)		493 (68.7)	500 (69.6)	
Performed	255 (32.0)	397 (30.8)		225 (31.3)	218 (30.4)	
Radiotherapy			<0.001			0.914
Not performed	429 (53.9)	902 (70.1)		428 (59.6)	426 (59.3)	
Performed	367 (46.1)	385 (29.9)		290 (40.4)	292 (40.7)	
Lymphedema			0.005			0.866
No	694 (87.2)	1172 (91.1)		638 (88.9)	640 (89.1)	
Yes	102 (12.8)	115 (8.9)		80 (11.1)	78 (10.9)	
Axillary surgery			<0.001			0.196
SLNB only	261 (32.8)	549 (42.7)		246 (34.3)	223 (31.1)	
ALND	535 (67.2)	738 (57.3)		472 (65.7)	495 (68.9)	
Diabetes			0.556			0.423
No	748 (94.0)	1201 (93.3)		674 (93.9)	861 (94.9)	
Yes	48 (6.0)	86 (6.7)		44 (6.1)	37 (5.1)	
Dyslipidemia			0.936			0.418
No	595 (74.8)	960 (64.6)		543 (75.6)	556 (77.4)	
Yes	201 (25.2)	327 (25.4)		175 (24.4)	162 (22.6)	
Autoimmune disease *			0.856			0.739
No	698 (87.7)	1132 (88.0)		635 (88.4)	639 (89.0)	
Yes	98 (12.3)	155 (12.0)		83 (11.6)	79 (11.0)	
Rheumatoid arthritis			0.537			0.889
No	762 (95.7)	1239 (96.3)		691 (96.2)	692 (96.4)	
Yes	34 (4.3)	48 (3.7)		27 (3.8)	26 (3.6)	
Lupus erythematous			0.639			0.500
No	794 (99.8)	1285 (99.8)		716 (99.7)	718 (100.0)	
Yes	2 (0.2)	2 (0.2)		2 (0.3)	0 (0.0)	
Systemic sclerosis			-			-
No	796 (100.0)	1287 (100.0)		718 (100.0)	718 (0.0)	
Yes	0 (0.0)	0 (0.0)		0 (0.0)	0 (0.0)	
Sicca syndrome			>0.999			>0.999
No	796 (100.0)	1286 (99.9)		718 (100.0)	717 (99.9)	
Yes	0 (0.0)	1 (0.1)		0 (0.0)	1 (0.1)	
Psoriasis			0.724			0.488
No	784 (98.5)	1270 (98.7)		707 (98.5)	710 (98.9)	
Yes	12 (1.5)	17 (1.3)		11 (1.5)	8 (1.1)	
Behcet’s disease			>0.999			>0.999
No	796 (100.0)	1286 (99.9)		718 (100.0)	717 (99.9)	
Yes	0 (0.0)	1 (0.1)		0 (0.0)	1 (0.1)	
Autoimmune hepatitis			-			-
No	796 (100.0)	1287 (100.0)		718 (100.0)	718 (0.0)	
Yes	0 (0.0)	0 (0.0)		0 (0.0)	0 (0.0)	
Autoimmune thyroiditis			0.506			0.807
No	787 (98.9)	1268 (98.5)		709 (98.8)	710 (98.9)	
Yes	9 (1.1)	19 (1.5)		9 (1.2)	8 (1.1)	
Autoimmune adrenalitis			-			-
No	796 (100.0)	1287 (100.0)		718 (100.0)	718 (0.0)	
Yes	0 (0.0)	0 (0.0)		0 (0.0)	0 (0.0)	
Systemic connective tissue disorder			0.382			>0.999
No	795 (99.9)	1287 (100.0)		717 (99.9)	718 (100.0)	
Yes	1 (0.1)	0 (0.0)		1 (0.1)	0 (0.0)	
Atopic dermatitis			0.263			0.280
No	757 (95.1)	1209 (93.9)		686 (95.5)	677 (94.3)	
Yes	39 (4.9)	78 (6.1)		32 (4.5)	41 (5.7)	
Vitiligo			>0.999			0.625
No	793 (99.6)	1283 (99.7)		715 (99.6)	717 (99.9)	
Yes	3 (0.4)	4 (0.3)		3 (0.4)	1 (0.1)	
Steroid medication			0.185			0.519
No	757 (95.1)	1206 (93.7)		684 (95.3)	689 (96.0)	
Yes	39 (4.9)	81 (6.3)		34 (4.7)	29 (4.0)	

CCI, Charlson Comorbidity index; SD, standard deviation; HER2, human epidermal growth factor receptor 2; SLNB, sentinel lymph node biopsy; ALND, axillary lymph node dissection. * Autoimmune disease is defined as having one of the following autoimmune diseases: rheumatoid arthritis, lupus erythematous, systemic sclerosis, Sicca syndrome, psoriasis, Behcet’s disease, autoimmune hepatitis, autoimmune thyroiditis, autoimmune adrenalitis, systemic connective tissue disorder, atopic dermatitis, or vitiligo.

**Table 2:** Comparison of clinical characteristics of breast cancer patients undergoing TEI reconstruction after total mastectomy according to chemotherapy type.

	Before Matching			After Matching		
	Patients Receiving Neoadjuvant Chemotherapy, n = 921 (%)	Patients Receiving Adjuvant Chemotherapy, n = 1299 (%)	*p* Value	Patients Receiving Neoadjuvant Chemotherapy, n = 767 (%)	Patients Receiving Adjuvant Chemotherapy, n = 767 (%)	*p* Value
Capsulectomy only			0.088			0.441
Not performed	817 (88.7)	1181 (90.9)		687 (89.6)	696 (90.7)	
Performed	104 (11.3)	118 (9.1)		80 (10.4)	71 (9.3)	
Both capsulectomy and implant change			<0.001			-
Not performed	902 (97.9)	1299 (100)		767 (100)	767 (100)	
Performed	19 (2.1)	0		0	0	
Age (year)			<0.001			0.945
20–29	31 (3.4)	18 (1.4)		11 (1.4)	15 (2.0)	
30–39	229 (24.9)	220 (16.9)		155 (20.2)	158 (20.6)	
40–49	383 (41.6)	596 (45.9)		345 (45.4)	351 (45.8)	
50–59	233 (25.3)	362 (27.9)		209 (27.3)	198 (25.8)	
60–69	42 (4.6)	96 (7.4)		41 (5.4)	43 (5.6)	
70–79	3 (0.3)	7 (0.5)		3 (0.4)	2 (0.3)	
CCI (Weight number, mean ± SD)	4.29 ± 2.73	3.66 ± 2.27	<0.001	3.98 ± 2.51	3.92 ± 2.470	0.644
Endocrine therapy			0.001			0.408
Not performed	268 (29.1)	300 (23.1)		197 (25.7)	183 (23.9)	
Performed	653 (70.9)	999 (76.9)		570 (74.3)	584 (76.1)	
HER2-target therapy			0.479			0.779
Not performed	651 (70.7)	900 (69.3)		544 (70.9)	539 (70.3)	
Performed	270 (29.3)	399 (30.7)		223 (29.1)	228 (29.7)	
Radiotherapy			<0.001			0.958
Not performed	542 (58.9)	912 (70.2)		493 (64.3)	492 (64.2)	
Performed	379 (41.1)	387 (29.8)		274 (35.7)	275 (35.9)	
Lymphedema			0.046			0.799
No	815 (88.5)	1183 (91.1)		691 (90.1)	688 (89.7)	
Yes	106 (11.5)	116 (8.9)		76 (9.9)	79 (10.3)	
Axillary surgery			0.393			0.172
SLNB only	381 (41.4)	561 (43.2)		308 (40.2)	282 (36.8)	
ALND	540 (58.6)	738 (56.8)		459 (59.8)	485 (63.2)	
Diabetes			0.707			0.172
No	863 (93.7)	1212 (93.3)		718 (93.6)	722 (94.1)	
Yes	58 (6.3)	87 (6.7)		49 (6.4)	45 (5.9)	
Dyslipidemia			0.829			0.906
No	684 (74.3)	970 (74.7)		577 (75.2)	579 (75.5)	
Yes	237 (25.7)	329 (25.3)		190 (24.8)	188 (24.5)	
Autoimmune disease *			0.202			0.580
No	792 (86.0)	1141 (87.8)		680 (88.7)	673 (87.7)	
Yes	129 (14.0)	158 (12.2)		87 (11.3)	94 (12.3)	
Rheumatoid arthritis			0.499			0.444
No	881 (95.7)	1250 (96.2)		738 (96.2)	732 (95.4)	
Yes	40 (4.3)	49 (3.8)		29 (3.8)	35 (4.6)	
Lupus erythematous			0.24			-
No	917 (99.6)	1297 (99.9)		764 (99.6)	767 (100.0)	
Yes	4 (0.4)	2 (0.1)		3 (0.4)	0 (0.0)	
Systemic sclerosis			-			-
No	921 (100.0)	1299 (100.0)		767 (100.0)	767 (100.0)	
Yes	0 (0.0)	0 (0.0)		0 (0.0)	0 (0.0)	
Sicca syndrome			>0.999			
No	921 (100.0)	1298 (99.9)		767 (100.0)	767 (100.0)	
Yes	0 (0.0)	1 (0.1)		0 (0.0)	0 (0.0)	
Psoriasis			0.676			0.132
No	907 (98.5)	1282 (96.7)		756 (98.6)	762 (99.4)	
Yes	14 (1.5)	17 (1.3)		11 (1.4)	5 (0.6)	
Behcet’s disease			>0.999			>0.999
No	921 (100.0)	1298 (99.9)		767 (100.0)	766 (99.9)	
Yes	0 (0.0)	1 (0.1)		0 (0.0)	1 (0.1)	
Autoimmune hepatitis						-
No	921 (100.0)	1299 (100.0)		767 (100.0)	767 (100.0)	
Yes	0 (0.0)	0 (0.0)		0 (0.0)	0 (0.0)	
Autoimmune thyroiditis			0.698			0.668
No	908 (98.6)	1278 (98.4)		757 (98.7)	755 (98.4)	
Yes	13 (1.4)	21 (1.6)		10 (1.3)	12 (1.6)	
Autoimmune adrenalitis			-			-
No	921 (100.0)	1299 (100.0)		767 (100.0)	767 (100.0)	
Yes	0 (0.0)	0 (0.0)		0 (0.0)	0 (0.0)	
Systemic connective tissue disorder			0.415			>0.999
No	920 (99.9)	1299 (100.0)		766 (99.9)	767 (100.0)	
Yes	1 (0.1)	0 (0.0)		1 (0.1)	0 (0.0)	
Atopic dermatitis			0.777			0.108
No	863 (93.7)	1221 (94.0)		734 (95.7)	720 (93.9)	
Yes	58 (6.3)	78 (6.0)		33 (4.3)	47 (6.1)	
Vitiligo			>0.999			0.250
No	918 (99.7)	1295 (99.7)		764 (99.6)	767 (100.0)	
Yes	3 (0.3)	4 (0.3)		3 (0.4)	0 (0.0)	
Steroid medication			0.059			0.580
No	880 (95.6)	1217 (93.7)		729 (95.1)	734 (95.7)	
Yes	41 (4.4)	82 (6.3)		38 (4.9)	33 (4.3)	

CCI, Charlson Comorbidity index; SD, standard deviation; HER2, human epidermal growth factor receptor 2; SLNB, sentinel lymph node biopsy; ALND, axillary lymph node dissection. * Autoimmune disease is defined as having one of the following autoimmune diseases: rheumatoid arthritis, lupus erythematous, systemic sclerosis, Sicca syndrome, psoriasis, Behcet’s disease, autoimmune hepatitis, autoimmune thyroiditis, autoimmune adrenalitis, systemic connective tissue disorder, atopic dermatitis, or vitiligo.

**Table 3:** Risk of developing implant contracture in breast cancer patients according to the type of chemotherapy in the DTI reconstruction cohort using the Cox proportional hazard model.

	Before Matching						After Matching					
	Univariate Analysis		Multivariate Analysis Model 1 *		Multivariate Analysis Model 2 **		Univariate Analysis		Multivariate Analysis Model 1 *		Multivariate Analysis Model 2 **	
	HR (95% CIs)	*p* Value	HR (95% CIs)	*p* Value	HR (95% CIs)	*p* Value	HR (95% CIs)	*p* Value	HR (95% CIs)	*p* Value	HR (95% CIs)	*p* Value
Age (per 10 years)	1.239 (1.067–1.440)	0.005	1.253 (1.068–1.469)	0.006	1.254 (1.070–1.470)	0.005	1.146 (0.955–1.374)	0.143	1.157 (0.952–1.406)	0.142	1.163 (0.958–1.412)	0.127
CCI (Weight number)	1.033 (0.979–1.090)	0.004	1.004 (0.947–1.065)	0.886	1.007 (0.949–1.067)	0.824	0.999 (0.934–1.067)	0.967	0.973 (0.906–1.046)	0.460	0.976 (0.909–1.048)	0.511
Chemotherapy type		0.056		0.071		0.068		0.122		0.133		0.118
Neoadjuvant	reference		reference		Reference		reference		reference		reference	
Adjuvant	0.765 (0.581–1.007)		0.768 (0.577–1.023)		0.766 (0.575–1.020)		0.774 (0.559–1.071)		0.778 (0.561–1.079)		0.771 (0.556–1.068)	
Endocrine therapy		0.813		0.679		0.717		0.854		0.961		0.994
Not performed	reference		reference		reference		reference		reference		reference	
Performed	0.963 (0.703–1.318)		1.072 (0.772–1.489)		1.063 (0.765–1.476)		0.966 (0.665–1.402)		1.010 (0.682–1.495)		0.998 (0.675–1.477)	
HER2-target therapy		0.259		0.390		0.381		0.854		0.771		0.830
Not performed	reference		reference		reference		reference		reference		reference	
Performed	1.180 (0.885–1.573)		1.139 (0.846–1.533)		1.141 (0.849–1.534)		1.033 (0.729–1.464)		1.055 (0.734–1.516)		1.041 (0.725–1.494)	
Radiotherapy		0.004		0.006		0.006		0.011		0.008		0.008
Not performed	reference		reference		reference		reference		reference		reference	
Performed	1.504 (1.143–1.978)		1.505 (1.128–2.008)		1.505 (1.128–2.008)		1.526 (1.104–2.108)		1.569 (1.123–2.191)		1.566 (1.122–2.188)	
Lymphedema		<0.001		0.003		0.002		0.005		0.007		0.006
No	reference		reference		reference		reference		reference		reference	
Yes	1.907 (1.336–2.722)		1.778 (1.221–2.588)		1.800 (1.238–2.617)		1.812 (1.194–2.752)		1.840 (1.186–2.855)		1.842 (1.189–2.855)	
Axillary surgery		0.141		0.016		0.016		0.051		0.007		0.008
SLNB	reference		reference		reference		reference		reference		reference	
ALND	0.809 (0.610–1.073)		0.693 (0.515–0.933)		0.695 (0.517–0.935)		0.713 (0.507–1.002)		0.615 (0.431–0.876)		0.622 (0.437–0.885)	
Diabetes		0.192		0.546		0.558		0.291		0.280		0.302
No	reference		reference		reference		reference		reference		reference	
Yes	1.392 (0.847–2.286)		1.181 (0.689–2.023)		1.174 (0.686–2.012)		1.392 (0.753–2.574)		1.454 (0.737–2.870)		1.431 (0.725–2.823)	
Dyslipidemia		0.397		0.821		0.822		0.910		0.441		0.434
No	reference		reference		reference		reference		reference		reference	
Yes	1.142 (0.840–1.552)		0.963 (0.692–1.338)		0.963 (0.693–1.338)		0.978 (0.664–1.441)		0.849 (0.559–1.288)		0.847 (0.558–1.285)	
Autoimmune disease		0.345				0.619		0.740				0.914
No	reference				reference		reference				reference	
Yes	1.212 (0.813–1.806)				1.109 (0.738–1.666)		1.090 (0.657–1.807)				1.029 (0.614–1.723)	
Rheumatoid arthritis		0.212		0.482				0.722		0.910		
No	reference		reference				reference		reference			
Yes	1.472 (0.802–2.703)		1.252 (0.669–2.345)				1.160 (0.512–2.627)		0.949 (0.382–2.354)			
Lupus erythematous		0.384		0.501				0.132		0.116		
No	reference		reference				reference		reference			
Yes	2.394 (0.335–17.087)		1.994 (0.267–14.908)				4.550 (0.635–32.607)		5.836 (0.648–52.568)			
Systemic connective tissue disorder		0.262						0.278				
No	reference						reference					
Yes	4.918 (0.304–79.530)						4.672 (0.288–75.884)					
Sicca syndrome		0.308						0.327				
No	reference						reference					
Yes	4.248 (0.263–68.651)						4.027 (0.248–65.384)					
Psoriasis		0.290		0.315				0.512		0.615		
No	reference		reference				reference		reference			
Yes	0.346 (0.049–2.464)		0.365 (0.051–2.605)				0.518 (0.072–3.700)		0.603 (0.084–4.334)			
Behcet’s disease		0.308						0.327				
No	reference						reference					
Yes	4.248 (0.263–68.651)						4.027 (0.248–65.384)					
Autoimmune thyroiditis		0.735		0.537				0.635		0.596		
No	reference		reference				reference		reference			
Yes	0.786 (0.195–3.164)		0.642 (0.158–2.616)				0.621 (0.087–4.440)		0.586 (0.081–4.224)			
Atopic dermatitis		0.186		0.291				0.435		0.582		
No	reference		reference				reference		reference			
Yes	1.426 (0.843–2.413)		1.332 (0.782–2.266)				1.309 (0.667–2.569)		1.211 (0.612–2.398)			
Vitiligo		0.860						0.827				
No	reference						reference					
Yes	0.778 (0.048–12.573)						1.365 (0.084–22.126)					
Steroid medication		0.536		0.603		0.545		0.874		0.943		0.913
No	reference		reference		reference		reference		reference		reference	
Yes	1.187 (0.690–2.042)		1.157 (0.668–2.004)		1.184 (0.685–2.047)		1.063 (0.498–2.272)		0.972 (0.452–2.091)		1.958 (0.446–2.060)	

HR, hazard ratio; CI, confidence interval; CCI, Charlson Comorbidity index; HER2, human epidermal growth factor receptor 2, SLNB, sentinel lymph node biopsy; ALND, axillary lymph node dissection. * Multivariate analysis model 1: If any of the autoimmune diseases, such as rheumatoid arthritis, lupus erythematous, or systemic sclerosis, etc., are present, the variable is defined as comprehensive autoimmune disease. ** Multivariate analysis model 2: Analysis including each autoimmune disease as a variable.

**Table 4:** Risk of developing implant contracture in breast cancer patients according to the type of chemotherapy in the TEI cohort using the Cox proportional hazard model.

	Before Matching						After Matching					
	Univariate Analysis		Multivariate Analysis Model 1 *		Multivariate Analysis Model 2 **		Univariate Analysis		Multivariate Analysis Model 1 *		Multivariate Analysis Model 2 **	
	HR (95% CIs)	*p* Value	HR (95% CIs)	*p* Value	HR (95% CIs)	*p* Value	HR (95% CIs)	*p* Value	HR (95% CIs)	*p* Value	HR (95% CIs)	*p* Value
Age (per 10 years)	1.219 (1.055–1.407)	0.007	1.242 (1.066–1.447)	0.006	1.242 (1.066–1.446)	0.005	1.179 (0.985–1.412)	0.073	1.190 (0.977–1.449)	0.084	1.193 (0.980–1.453)	0.079
CCI (Weight number)	1.041 (0.990–1.095)	0.118	1.012 (0.958–1.069)	0.675	1.014 (0.960–1.071)	0.623	1.007 (0.945–1.074)	0.825	0.982 (0.916–1.052)	0.598	0.984 (0.918–1.054)	0.639
Chemotherapy type		0.020		0.02		0.025		0.213		0.214		0.212
Neoadjuvant	reference		reference		reference		reference		reference		reference	
Adjuvant	0.730 (0561–0.951)		0.728 (0.557–0.951)		0.731 (0.556–0.961)		0.816 (0.593–1.124)		0.816 (0.592–1.124)		0.815 (0.592–1.123)	
Endocrine therapy		0.760		0.742		0.785		0.498		0.365		0.366
Not performed	reference		reference		reference		reference		reference		reference	
Performed	0.954 (0.706–1.290)		1.054 (0.770–1.442)		1.044 (0.764–1.429)		1.143 (0.777–1.681)		1.206 (0.805–1.806)		1.205 (0.804–1.805)	
HER2-target therapy		0.496		0.639		0.624		0.485		0.558		0.566
Not done	reference		reference		reference		reference		reference		reference	
Done	1.103 (0.832–1.460)		1.072 (0.802–1.432)		1.075 (0.805–1.435)		0.880 (0.615–1.260)		0.895 (0.618–1.296)		0.898 (0.620–1.298)	
Radiotherapy		0.003		0.005		0.004		0.028		0.012		0.013
Not performed	reference		reference		reference		reference		reference		reference	
Performed	1.491 (1.144–1.943)		1.497 (1.134–1.978)		1.498 (1.134–1.979)		1.432 (1.039–1.974)		1.531 (1.098–2.134)		1.527 (1.095–2.129)	
Lymphedema		<0.001		0.002		0.002		0.026		0.023		0.021
No	reference		reference		reference		reference		reference		reference	
Yes	1.887 (1.336–2.667)		1.775 (1.233–2.557)		1.801 (1.252–2.590)		1.639 (1.060–2.536)		1.703 (1.078–2.691)		1.710 (1.083–2.701)	
Axillary surgery		0.046		0.002		0.002		0.003		<0.001		<0.001
SLNB	reference		reference		reference		reference		reference		reference	
ALND	0.761 (0.581–0.996)		0.643 (0.485–0.853)		0.645 (0.487–0.855)		0.604 (0.436–0.837)		0.523 (0.373–0.734)		0.516 (0.368–0.725)	
Diabetes		0.123		0.454		0.461		0.065		0.104		0.100
No	reference		reference		reference		reference		reference		reference	
Yes	1.447 (0.904–2.316)		1.215 (0.730–2.023)		1.212 (0.728–2.018)		1.678 (0.968–2.908)		1.660 (0.900–3.062)		1.672 (0.907–3.083)	
Dyslipidemia		0.405		0.785		0.792		0.837		0.525		0.555
No	reference		reference		reference		reference		reference		reference	
Yes	1.134 (0.843–1.525)		0.957 (0.696–1.315)		0.958 (0.697–1.317)		1.040 (0.717–1.507)		0.877 (0.585–1.315)		0.885 (0.590–1.327)	
Autoimmune disease		0.343				0.657		0.354				0.227
No	reference				reference		reference				reference	
Yes	1.202 (0.822–1.757)				1.092 (0.741–1.610)		0.764 (0.433–1.350)				0.699 (0.391–1.249)	
Rheumatoid arthritis		0.201		0.503				0.970		0.792		
No	reference		reference				reference		reference			
Yes	1.462 (0.817–2.617)		1.228 (0.673–2.240)				0.984 (0.435–2.228)		0.894 (0.390–2.052)			
Lupus erythematous		0.483		0.610				0.513				
No	reference		reference				reference					
Yes	2.021 (0.284–14.405)		1.686 (0.227–12.535)				2.547 (0.155–41.858)					
Systemic connective tissue disorder		0.275										
No	reference											
Yes	4.717 (0.292–76.216)											
Sicca syndrome		0.320										
No	reference											
Yes	4.103 (0.254–66.270)											
Psoriasis		0.251		0.272				0.411				
No	reference		reference				reference					
Yes	0.317 (0.045–2.255)		0.333 (0.047–2.371)				0.312 (0.019–5.030)					
Behcet’s disease		0.320										
No	reference											
Yes	4.103 (0.254–66.270)											
Autoimmune thyroiditis		0.582		0.421				0.354				
No	reference		reference				reference					
Yes	0.677 (0.168–2.720)		0.563 (0.139–2.284)				0.268 (0.017–4.347)					
Atopic dermatitis		0.149		0.241				0.970		0.831		
No	reference		reference				reference		reference			
Yes	1.440 (0.878–2.363)		1.349 (0.818–2.224)				0.985 (0.461–2.105)		0.920 (0.427–1.981)			
Vitiligo		0.837						0.680				
No	reference						reference					
Yes	0.746 (0.046–12.041)						1.798 (0.111–29.162)					
Steroid medication		0.666		0.703		0.659		0.793		0.955		0.925
No	reference		reference		reference		reference		reference		reference	
Yes	1.127 (0.656–1.936)		1.112 (0.644–1.921)		1.131 (0.655–1.951)		1.100 (0.540–2.242)		1.021 (0.497–2.098)		1.035 (0.504–2.126)	

HR, hazard ratio; CI, confidence interval; CCI, Charlson Comorbidity index; HER2, human epidermal growth factor receptor 2, SLNB, sentinel lymph node biopsy; ALND, axillary lymph node dissection. * Multivariate analysis model 1: If any of the autoimmune diseases, such as rheumatoid arthritis, lupus erythematous, or systemic sclerosis, etc., are present, the variable is defined as comprehensive autoimmune disease. ** Multivariate analysis model 2: Analysis including each autoimmune disease as a variable.

## Data Availability

All data generated or analyzed during this study are included in this research article and Supplementary Information files. However, the original data are prohibited from being exported outside due to HIRA’s data protection policy.

## Supplementary Materials

The following supporting information can be downloaded at: https://www.mdpi.com/article/10.3390/cancers17122053/s1, Table S1. Behavior codes; Table S2. Drug prescription codes; Table S3. Diagnostic codes; Table S4. Charlson Comorbidity Index; Table S5. Comparison of clinical characteristics of breast cancer patients undergoing DTI reconstruction after total mastectomy according to chemotherapy duration; Table S6. Comparison of clinical characteristics of breast cancer patients undergoing TEI reconstruction after total mastectomy according to chemotherapy duration; Table S7. Risk of developing implant contracture in breast cancer patients according to the duration of chemotherapy in the DTI cohort using the Cox proportional hazard model; Table S8. Risk of developing implant contracture in breast cancer patients according to the duration of chemotherapy in the TEI cohort using the Cox proportional hazard model; Figure S1. Schematic diagram illustrating the patient’s enrollment and exclusion criteria; Figure S2. Cumulative incidence of capsular contracture in breast cancer patients undergoing DTI surgery by chemotherapy duration; Figure S3. Cumulative incidence of capsular contracture in breast cancer patients undergoing TEI surgery by chemotherapy duration.

## Abbreviations

The following abbreviations are used in this manuscript:

IBBR	Implant-based breast reconstruction
HIRA	Health Insurance Review and Assessment Service
IRB	Institutional Review Board
DCIS	Ductal carcinoma in situ
ICD-10	International Classification of Disease, 10th revision
DTI	Direct-to-implant
TEI	Tissue-expander insertion
HER2	Human epidermal growth factor receptor 2
CCI	Charlson Comorbidity index
DM	Diabetes mellitus
ALND	Axillary lymph node dissection
