# Supplementary material for: Impact of Chemotherapy on Implant-Based Breast Reconstruction in Breast Cancer Patients: A Nationwide, Retrospective, Cohort Study
Source: Cancers (Basel). 2025 Jun 19;17(12):2053. doi: 10.3390/cancers17122053 (PMC12190455; doi:10.3390/cancers17122053)
Supplement: Supplementary file 1 [file cancers-17-02053-s001.zip › cancers-3661444-supplementary.pdf]

Table S1. Behavior codes

Code number	Behavior code
Surgical codes	
N7135	radical mastectomy including modified radical mastectomy and radical wide excision with axillary surgery
N7138	total mastectomy with axillary surgery
N7139	total mastectomy without axillary surgery
N7148	Insertion of breast tissue expander, including subsequent expansion
N7149	Immediate insertion of permanent breast implant following mastectomy
N7150	Insertion of permanent breast implant following breast tissue expansion
N7151	Breast capsulectomy (breast capsulorrhaphy, capsulotomy, capsular flap)
Radiation codes	
HD010	For Teletherapy Single Port
HD011	For Teletherapy Paralled Opposed Ports
HD012	For Teletherapy Non-Paralled Opposed Ports, more than 3 Ports
HD013	Computerized Radiotherapy Planning Single Port
HD014	Computerized Radiotherapy Planning Paralled Opposed Ports
HD015	Computerized Radiotherapy Planning Non-Paralled Opposed Ports, more than 3 Ports
HD016	Computerized Radiotherapy Planning Rotational Therapy
HD017	Computerized Radiotherapy Planning Intra-Operative Radiation Therapy
HD018	Computerized Radiotherapy Planning 3-Dimentional Conformal Radiation Therapy
HD019	Computerized Radiotherapy Planning Stereotactic Radiosurgery and Radiotherapy
HD020	Computerized Radiotherapy Planning Proton Therapy Planning
HD021	For Brachytherapy Mold Therapy
HD022	For Brachytherapy Intracavitary or Intraluminal Therapy
HD023	For Brachytherapy Interstitial Therapy
HD031	Design and Construction of Therapeutic Devices Shielding Block
HD032	Design and Construction of Therapeutic Devices Compensator or Bolus
HD033	Design and Construction of Therapeutic Devices Immobilization Devices
HD041	Computerized Radiotherapy Planning Intensity Modulated Radiation Therapy Planning
HD051	Teletherapy Low Energy Single Port
HD052	Teletherapy Middle Energy Single Port
HD053	Teletherapy High Energy Single Port
HD054	Teletherapy Low Energy Paralled Opposed Ports
HD055	Teletherapy Middle Energy Paralled Opposed Ports
HD056	Teletherapy High Energy Paralled Opposed Ports
HD057	Rotational Irradiation Low Energy
HD058	Rotational Irradiation Middle Energy
HD059	Rotational Irradiation High Energy
HD061	3-Dimensional Conformal Therapy Unsealed Source
HD410	For Teletherapy Single Port
HD411	For Teletherapy Paralled Opposed Ports
HD412	For Teletherapy Non-Paralled Opposed Ports, more than 3 Ports
HD413	Computerized Radiotherapy Planning Single Port
HD414	Computerized Radiotherapy Planning Paralled Opposed Ports
HD415	Computerized Radiotherapy Planning Non-Paralled Opposed Ports, more than 3 Ports
HD416	Computerized Radiotherapy Planning Rotational Therapy
HD418	Computerized Radiotherapy Planning 3-Dimentional Conformal Radiation Therapy
HD419	Computerized Radiotherapy Planning Stereotactic Radiosurgery and Radiotherapy
HD420	Computerized Radiotherapy Planning Proton Therapy Planning
HD441	Computerized Radiotherapy Planning Intensity Modulated Radiation Therapy Planning
HZ271	Intensity Modulated Radiation Therapy

Table S2. Drug prescription codes

Drugs	*Codes
**Endocrine medication**	
**Tamoxifen**	234501ATB, 234502ATB
**Aromatase inhibitor**	
**Letrozole**	182201ATB
**Anastrozole**	109001ATB
**Exemestane**	358401ATB
**GnRH analogues**	202BIJ, 167201BIJ, 182602BIJ, 182604BIJ, 182611BIJ, 220603BIJ 244902BIJ 244901BIJ 244930BIJ 467501BIJ 467502BIJ
**Chemotherapy**	
**Taxane**	148301BIJ, 148302BIJ, 148303BIJ, 148304BIJ, 148305BIJ, 148306BIJ, 148309BIJ, 148310BIJ, 148338BIJ, 148339BIJ, 148340BIJ, 148341BIJ, 148342BIJ, 148344BIJ, 148345BIJ, 148346BIJ, 148348BIJ, 148349BIJ, 148350BIJ, 148351BIJ
**Doxorubicin**	149401BIJ, 149402BIJ, 149403BIJ, 149404BIJ, 149405BIJ, 149406BIJ, 149430BIJ, 149431BIJ, 149432BIJ, 149433BIJ, 149434BIJ, 149435BIJ
**Cyclophosphamide**	139001ATB, 139003BIJ, 139004BIJ, 139005BIJ
**Fluorouracil**	161401BIJ, 161402BIJ, 161404BIJ, 161430BIJ, 161431BIJ, 161432BIJ
**Methotrexate**	192101ATB, 192102BIJ, 192103BIJ, 192104BIJ, 192105BIJ, 192107ATB, 192107BIJ, 192108BIJ, 192109BIJ, 192110BIJ, 192111BIJ, 192132BIJ, 192134BIJ, 192136BIJ, 192138BIJ, 192139BIJ, 192140BIJ, 192141BIJ, 192142BIJ, 192143BIJ, 192144BIJ
**Carboplatin**	123703BIJ, 123730BIJ, 123701BIJ, 123731BIJ, 123702BIJ, 123732BIJ, 123707BIJ, 123733BIJ, 123708BIJ, 123735BIJ, 123704BIJ, 123734BIJ, 123706BIJ, 123736BIJ
**Capecitabine**	122702ATB, 122701ATB
**HER2-target therapy**	
**Trastuzumab**	242802BIJ, 242801BIJ, 626001BIJ, 626002BIJ, 242803BIJ, 242830BIJ
**Pertuzumab**	624601BIJ
**Neratinib**	703301ATB
**TDM-1**	626001BIJ, 626002BIJ
**Other target therapy**	
**Olaparib**	643501ACH, 643502ATB, 643503ATB
**Talazoparib**	691001ACH, 691002ACH
**Pembrolizumab**	639001BIJ
**Steroid medication**	
**Hydrocortisone**	171201BIJ
**Fludrocortisone**	160201ATB
**Dexamethasone**	142232BIJ, 142201BIJ, 141901ATB, 141903ATB
**Prednisolone**	217001ATB, 217003ASY, 217034ASY, 217035ASY, 217302BIJ, 217030ASY
**Methylprednisolone**	193302ATB, 193601BIJ, 193604BIJ, 193502BIJ, 193530BIJ, 193531BIJ, 193602BIJ, 193305ATB, 193603BIJ
**Budesonide**	119501CMS, 119405CLQ, 119436CLQ, 119404CSI, 119533CSI, 119435CLQ, 119404CSI, 119403CAE, 119502CSI, 119530CSI, 119505CSI, 119506CSI, 119532CSI, 119406ACH, 119438CAE
**Ciclesonide**	510030CSI, 497131CSI, 497130CSI, 497102CSI, 497101CSI, 510001CSI
**Fluticasone**	162238CSI, 162206CSS, 162230CSS, 162231CSS, 162205CSI, 162233CSI, 162202CSI, 162235CSI, 162204CSI, 162236CSI, 162232CSI, 500401CSI, 500430CSI, 500431CSI, 500432CSI, 162207CSI
**Mometaosine+Furoate**	361301CSI, 361331CSI

*Drug claim codes at National Health Insurance Service

GnRh, gonadotropin releasing hormone; HER2, human epidermal growth factor receptor 2

Table S3. Diagnostic codes

Classification	Diagnosis	ICD-10 code
**Diabetes Mellitus**		E10, E11, E12, E13, E14
**Dyslipidemia**		E78
**Lymphedema**		I890, I972
**Autoimmune disease**	**Rheumatoid arthritis**	M05, M05.0, M05.1, M05.3, M05.8, M05.9, M06.0, M06.1, M06.2, M06.3, M06.4, M06.8, M08.0, M08.1, M08.2, M08.3, M08.4, M08.8, M08.9, M09.0, M09.1, M09.2, M09.8
	**Lupus Erythematous**	L93.0, L93.1, L93.2, M32, M32.0, M32.1, M32.8, M32.9
	**Systemic sclerosis**	L94.0, L94.1, L94.2, L94.3, L94.4, L94.5, L94.6, L94.8, L94.9, M34, M34.0, M34.1, M34.2, M34.8, M34.9
	**Sicca syndrome**	M35.0, M35.1, M35.2, M35.3, M35.4, M35.5, M35.6, M35.7, M35.8, M35.9
	**psoriasis**	M07.0, M07.1, M07.2, M07.3, M07.4, M07.5, M07.6, L40, L40.0, L40.1, L40.2, L40.3, L40.4, L40.5, L40.8, L40.9
	**Behcet disease**	M35.2, M35.3, M35.4, M35.5, M35.6, M35.7, M35.8, M35.9
	**Autoimmune hepatitis**	K 75.4
	**Autoimmune thyroiditis**	E06.3
	**Autoimmune adrenalitis**	E27.1
	**Systemic connective tissue disorders**	M30, M30.0, M30.1, M30.2, M30.3, M30.8, M31, M31.0, M31.1, M31.3, M31.4, M31.5, M31.6, M31.7, M31.8, M31.9, M33, M33.0, M33.1, M33.2, M33.9
	**Atopic dermatitis**	L20, L20.0, L20.8, L20.9
	**Vitiligo**	L80

Table S4. Charlson Comorbidity Index

Comorbidity	ICD-10 code	Updated Weight
Myocardial infarction	I21.x, I22.x, I25.2	0
Congestive heart failure	I09.9, I11.0, I13.0, I13.2, I25.5, I42.0, I42.5-I42.9, I43.x, I50.x, P29.0	2
Peripheral vascular disease	I70.x, I71.x, I73.1, I73.8, I73.9, I77.1, I79.0, I79.2, K55.1, K55.8, K55.9, Z95.8, Z95.9	0
Cerebrovascular disease	G45.x, G46.x, H34.0, I60.x, I69.x	0
Dementia	F00.x-F03.x, F05.1, G30.x, G31.1	2
Chronic pulmonary disease	I27.8, I27.9, J40.x-J47.x, J60.x-J67.x, J68.4, J70.1, J70.3	1
Rheumatologic disease	M05.x, M06.x, M31.5, M32.x, M34.x, M35.1, M35.3, M36.0	1
Peptic ulcer disease	K25.x-K28.x	0
Mild liver disease	B18.x, K70.0-K70.3, K70.9, K71.3-71.5, K71.7, K73.x, K74.x, K76.0, K76.2-K76.4, K76.8, K76.9, KZ94.4	2
Diabetes without chronic complication	E10.0, E10.1, E10.6, E10.8, E10.9, E11.0, E11.1, E11.6, E11.8, E11.9, E12.0, E12.1, E12.6, E12.8, E12.9, E13.0, E13.1, E13.6, E13.8, E13.9, E14.0, E14.1, E14.8, E14.9	2
Diabetes with chronic complication	E10.2-E10.5, E10.7, E11.2-E11.5, E11.7, E12.2-E12.5, E12.7, E13.2-E13.5, E13.7, E14.2-E14.5, E14.7	1
Hemiplegia or paraplegia	G04.1, G11.4, G80.1, G80.2, G81.x, G82.x, G83.0-G83.4, G83.9	2
Renal disease	I12.0, I13.1, N03.2-N03.7, N05.2-N05.7, N18.x, N19.x, N25.0, Z49.0-Z49..2, Z94.0, Z99.2	1
Any malignancy including leukemia and lymphoma	There are no eligible patients in this study	2
Moderate or severe liver disease	I185.0, I85.9, I86.4, I98.2, K70.4, K71.1, K72.1, K72.9, K76.5, K76.6, K76.7	4
Metastatic solid tumor	There are no eligible patients in this study	6
Acquired immune deficiency syndrome/human immune deficiency virus	B20.x-B22.x, B24.x	4

Table S5. Comparison of clinical characteristics of breast cancer patients undergoing DTI reconstruction after total mastectomy according to chemotherapy duration

	Before matching			After matching		
	**Within 4-cycles of chemotherapy (within 12 week), n=991 (%)**	**More than 5-cycles of chemotherapy (over 12 week), n=1,092 (%)**	***P* value**	**Within 4-cycles of chemotherapy (within 12 week), n=677 (%)**	**More than 5-cycles of chemotherapy (over 12 week), n=677 (%)**	***P* value**
**Capsulectomy only**			0.883			0.214
**Not done**	894 (90.2)	983 (90.0)		599 (88.5)	613 (90.6)	
**Done**	97 (9.8)	109 (10.0)		78 (11.5)	64 (9.5)	
**Age (year)**			0.042			0.998
**20-29**	13 (1.3)	27 (2.5)		12 (1.8)	12 (1.8)	
**30-39**	173 (17.5)	228 (20.9)		125 (18.5)	122 (18.0)	
**40-49**	439 (44.3)	483 (44.2)		309 (45.6)	318 (47.0)	
**50-59**	285 (28.8)	289 (26.5)		182 (26.9)	179 (26.4)	
**60-69**	75 (7.6)	61 (5.6)		45 (6.7)	42 (6.2)	
**70-79**	6 (0.6)	4 (0.4)		4 (0.6)	4 (0.6)	
**CCI (Weight number, mean±SD)**	3.53 ± 2.16	4.13 ± 2.60	<0.001	3.63 ± 2.24	3.67 ± 2.19	0.695
**Endocrine therapy**			0.871			0.795
**Not done**	249 (25.1)	271 (24.8)		155 (22.9)	151 (22.3)	
**Done**	742 (74.9)	821 (75.2)		522 (77.1)	526 (77.7)	
**HER2-target therapy**			0.791			0.627
**Not done**	678 (68.4)	753 (69.0)		485 (71.6)	493 (72.8)	
**Done**	313 (31.6)	339 (31.0)		192 (28.4)	184 (27.2)	

**Radiotherapy**			<0.001			0.952
**Not done**	797 (80.4)	534 (48.9)		490 (72.4)	491 (72.5)	
**Done**	194 (19.6)	558 (51.1)		187 (27.6)	186 (27.5)	
**Lymph edema**			<0.001			0.924
**No**	923 (93.1)	943 (86.4)		616 (91.0)	617 (91.1)	
**Yes**	68 (6.9)	149 (13.6)		61 (9.0)	60 (8.9)	
**Axillary surgery**			<0.001			0.864
**SLNB only**	503 (50.8)	307 (28.1)		230 (34.0)	233 (34.4)	
**ALND**	488 (49.2)	785 (71.9)		447 (66.0)	444 (65.6)	
**Diabetes**			0.502			0.722
**No**	931 (94.0)	1018 (93.2)		641 (94.7)	638 (94.2)	
**Yes**	60 (6.0)	74 (6.8)		36 (5.3)	39 (5.8)	
**Dyslipidemia**			0.532			0.751
**No**	746 (75.3)	809 (74.1)		515 (76.1)	510 (75.3)	
**Yes**	245 (24.7)	283 (25.9)		162 (23.9)	167 (24.7)	
**Autoimmune disease***			0.751			0.863
**No**	873 (88.1)	957 (87.6)		607 (88.8)	603 (89.1)	
**Yes**	118 (11.9)	135 (12.4)		76 (11.2)	74 (10.9)	
**Rheumatoid arthritis**			0.650			0.458
**No**	954 (96.3)	1047 (95.9)		656 (96.9)	651 (96.2)	
**Yes**	37 (3.7)	45 (4.1)		21 (3.1)	26 (3..8)	
**Lupus erythematous**			>.999			>.999
**No**	989 (99.8)	1090 (99.8)		676 (99.9)	676 (99.9)	
**Yes**	2 (0.2)	2 (0.2)		1 (0.1)	1 (0.1)	
**Systemic sclerosis**			-			-
**No**	991 (100.0)	1092 (100.0)		677 (100.0)	677 (100.0)	

**Yes**	0 (0.0)	0 (0.0)		0 (0.0)	0 (0.0)	
**Sicca syndrome**			>.999			>.999
**No**	991 (100.0)	1091 (99.9)		677 (100.0)	676 (99.9)	
**Yes**	0 (0.0)	1 (0.1)		0 (0.0)	1 (0.1)	
**Psoriasis**			0.939			0.807
**No**	977 (98.6)	1077 (98.6)		669 (98.8)	668 (98.7)	
**Yes**	14 (1.4)	15 (1.4)		8 (1.2)	9 (1.3)	
**Behcet's disease**			>.999			-
**No**	991 (100.0)	1091 (99.9)		677 (100.0)	677 (100.0)	
**Yes**	0 (0.0)	1 (0.1)		0 (0.0)	0 (0.0)	
**Autoimmune hepatitis**			-			-
**No**	991 (100.0)	1092 (100.0)		677 (100.0)	677 (100.0)	
**Yes**	0 (0.0)	0 (0.0)		0 (0.0)	0 (0.0)	
**Autoimmune thyroiditis**			0.100			0.807
**No**	982 (99.1)	1073 (98.3)		669 (98.8)	668 (98.7)	
**Yes**	9 (0.9)	19 (1.7)		8 (1.2)	9 (1.3)	
**Autoimmune adrenalitis**			-			-
**No**	991 (100.0)	1092 (100.0)		677 (100.0)	677 (100.0)	
**Yes**	0 (0.0)	0 (0.0)		0 (0.0)	0 (0.0)	
**Systemic connective tissue disorder**			0.476			>.999
**No**	990 (99.9)	1092 (100.0)		676 (99.9)	677 (100.0)	
**Yes**	1 (0.1)	0 (0.0)		1 (0.1)	0 (0.0)	
**Atopic dermatitis**			0.409			0.108
**No**	931 (94.0)	1035 (94.8)		636 (93.9)	949 (95.9)	
**Yes**	60 (6.0)	57 (5.2)		41 (6.1)	28 (4.1)	

**Vitiligo**			0.456			0.687
**No**	989 (99.8)	1087 (99.5)		675 (99.7)	673 (99.4)	
**Yes**	2 (0.2)	5 (0.5)		2 (0.3)	4 (0.6)	
**Steroid medication**			0.864			0.811
**No**	933 (94.2)	1030 (94.3)		641 (94.7)	639 (94.4)	
**Yes**	58 (5.8)	62 (5.7)		36 (5.3)	38 (5.6)	

CCI, Charlson Comorbidity index; SD, standard deviation; HER2, human epidermal growth factor receptor 2; SLNB, sentinel lymph node biopsy; ALND, axillary lymph node dissection

* Autoimmune disease is defined as having one of the following autoimmune diseases: rheumatoid arthritis, lupus erythematous, systemic sclerosis, Sicca syndrome, psoriasis, Behcet's disease, autoimmune hepatitis, autoimmune thyroiditis, autoimmune adrenalitis, systemic connective tissue disorder, atopic dermatitis, or vitiligo

Table S6. Comparison of clinical characteristics of breast cancer patients undergoing TEI reconstruction after total mastectomy according to chemotherapy duration

	Before matching			After matching		
	**Within 4-cycles of chemotherapy (within 12 week), n=1,047 (%)**	**More than 5-cycles of chemotherapy (over 12 week), n=1,173 (%)**	***P* value**	**Within 4-cycles of chemotherapy (within 12 week), n=734 (%)**	**More than 5-cycles of chemotherapy (over 12 week), n=734 (%)**	***P* value**
**Capsulectomy only**			0.921			0.540
**Not done**	943 (90.0)	1,055(89.9)		658 (89.7)	665 (90.6)	
**Done**	104 (10.0)	118 (10.1)		76 (10.4)	69 (9.4)	
**Both capsulectomy and implant change**			0.657			0.795
**Not done**	1039 (99.2)	1162 (99.1)		726 (98.9)	727 (99.1)	
**Done**	8 (0.8)	11 (0.9)		8 (1.1)	7 (0.9)	
**Age (year)**			0.077			0.946
**20-29**	20 (1.9)	29 (2.5)		15 (2.0)	14 (1.9)	
**30-39**	190 (18.2)	259 (22.1)		150 (20.4)	150 (20.4)	
**40-49**	461 (44.0)	518 (44.2)		329 (44.8)	333 (45.4)	
**50-59**	295 (28.2)	300 (25.6)		185 (25.2)	192 (26.2)	
**60-69**	757 (7.2)	63 (5.4)		50 (6.8)	41 (5.6)	
**70-79**	6 (0.6)	4 (0.3)		5 (0.7)	4 (0.5)	
**CCI (Weight number, mean±SD)**	3.58 ± 2.20	4.23 ± 2.68	<0.001	3.76 ± 2.37	3.82 ± 2.34	0.634
**Endocrine therapy**			0.855			0.493
**Not done**	266 (25.4)	302 (25.8)		172 (23.4)	161 (21.9)	

**Done**	781 (74.6)	871 (74.2)		562 (76.6)	573 (78.1)	
**HER2-target therapy**			0.747			0.518
**Not done**	728 (69.5)	823 (70.2)		541 (73.7)	530 (72.2)	
**Done**	319 (30.5)	350 (29.8)		193 (26.3)	204 (27.8)	
**Radiotherapy**			<0.001			0.814
**Not done**	848 (81.0)	606 (51.7)		537 (73.2)	533 (72.6)	
**Done**	199 (19.0)	567 (48.3)		197 (26.8)	201 (27.4)	
**Lymph edema**			<0.001			0.925
**No**	978 (93.4)	1020 (87.0)		682 (92.9)	685 (93.3)	
**Yes**	69 (6.6)	153 (13.0)		52 (7.1)	49 (6.7)	
**Axillary surgery**			<0.001			0.490
**SLNB only**	559 (53.4)	383 (32.7)		292 (39.8)	305 (41.5)	
**ALND**	488 (46.6)	790 (67.3)		442 (60.2)	429 (58.4)	
**Diabetes**			0.451			0.660
**No**	983 (93.9)	1092 (93.1)		692 (94.3)	688 (93.7)	
**Yes**	64 (6.1)	81 (6.9)		42 (5.7)	46 (6.3)	
**Dyslipidemia**			0.286			0.581
**No**	791 (75.5)	863 (73.6)		564 (76.8)	555 (75.6)	
**Yes**	256 (54.5)	310 (26.4)		170 (23.8)	179 (24.4)	
**Autoimmune disease***			0.671			0.514
**No**	915 (87.4)	1018 (86.8)		653 (89.0)	645 (87.9)	
**Yes**	132 (12.6)	155 (13.2)		81 (11.0)	89 (12.1)	
**Rheumatoid arthritis**			0.669			0.680
**No**	1007 (96.2)	1124 (95.8)		708 (96.5)	705 (96.1)	
**Yes**	40 (3.8)	49 (4.2)		26 (3.5)	29 (3.9)	
**Lupus erythematous**			0.690			0.625

**No**	1045 (99.8)	1169 (99.7)		733(99.9)	731 (99.6)	
**Yes**	2 (0.2)	4 (0.3)		1 (0.1)	3 (0.4)	
**Systemic sclerosis**			-			-
**No**	1047 (100.0)	1173 (100.0)		734 (100.0)	734 (100.0)	
**Yes**	0 (0.0)	0 (0.0)		0 (0.0)	0 (0.0)	
**Sicca syndrome**			>.999			>.999
**No**	1047 (100.0)	1172 (99.9)		734 (100.0)	733 (99.9)	
**Yes**	0 (0.0)	1 (0.1)		0 (0.0)	1 (0.1)	
**Psoriasis**			0.891			0.807
**No**	1032 (98.6)	1157 (98.6)		726 (98.9)	725 (98.8)	
**Yes**	15 (1.4)	16 (1.4)		8 (1.1)	9 (1.2)	
**Behcet's disease**			>.999			>.999
**No**	1047 (100.0)	1172 (99.9)		734 (100.0)	733 (99.9)	
**Yes**	0 (0.0)	1 (0.1)		0 (0.0)	1 (0.1)	
**Autoimmune hepatitis**			-			-
**No**	1047 (100.0)	1173 (100.0)		734 (100.0)	734 (100.0)	
**Yes**	0 (0.0)	0 (0.0)		0 (0.0)	0 (0.0)	
**Autoimmune thyroiditis**			0.037			0.817
**No**	1037 (99.0)	1149 (97.9)		724 (98.6)	725 (98.8)	
**Yes**	10 (1.0)	24 (2.1)		10 (1.4)	9 (1.2)	
**Autoimmune adrenalitis**			-			-
**No**	1047 (100.0)	1173 (100.0)		734 (100.0)	734 (100.0)	
**Yes**	0 (0.0)	0 (0.0)		0 (0.0)	0 (0.0)	
**Systemic connective tissue disorder**			0.472			>.999
**No**	1046 (99.9)	1173 (100.0)		733 (99.9)	734 (100.0)	

**Yes**	1 (0.1)	0 (0.0)		1 (0.1)	0 (0.0)	
**Atopic dermatitis**			0.389			0.732
**No**	978 (9.34)	1106 (94.3)		695 (94.7)	692 (94.3)	
**Yes**	69 (6.6)	67 (5.7)		39 (5.3)	42 (5.7)	
**Vitiligo**			0.458			>.999
**No**	1045 (99.8)	1168 (99.6)		732 (99.7)	731 (99.6)	
**Yes**	2 (0.2)	5 (0.4)		2 (0.3)	3 (0.4)	
**Steroid medication**			0.711			0.548
**No**	987 (94.3)	1110 (94.6)		700 (95.4)	695 (94.7)	
**Yes**	60 (5.7)	63 (5.4)		34 (4.6)	39 (5.3)	

CCI, Charlson Comorbidity index; SD, standard deviation; HER2, human epidermal growth factor receptor 2; SLNB, sentinel lymph node biopsy; ALND, axillary lymph node dissection

* Autoimmune disease is defined as having one of the following autoimmune diseases: rheumatoid arthritis, lupus erythematous, systemic sclerosis, Sicca syndrome, psoriasis, Behcet’s disease, autoimmune hepatitis, autoimmune thyroiditis, autoimmune adrenalitis, systemic connective tissue disorder, atopic dermatitis, or vitiligo

Table S7. Risk of developing implant contracture in breast cancer patients according to the duration of chemotherapy in the DTI cohort using the Cox proportional hazard model

	Before matching						After matching					
	Univariate analysis		Multivariate analysis model 1*		Multivariate analysis model 2**		Univariate analysis		Multivariate analysis model 1*		Multivariate analysis model 2**	
	HR (95% CIs)	*P* value	HR (95% CIs)	*P* value	HR (95% CIs)	*P* value	HR (95% CIs)	*P* value	HR (95% CIs)	*P* value	HR (95% CIs)	*P* value
**Age (per 10-year)**	1.239 (1.067-1.440)	0.005	1.235 (1.054-1.448)	0.009	1.235 (1.055-1.447)	0.009	1.194 (0.996-1.432)	0.055	1.216 (1.004-1.474)	0.046	1.189 (0.981-1.442)	0.077
**CCI (Weight number)**	1.033 (0.979-1.090)	0.239	1.009 (0.951-1.070)	0.773	1.011 (0.954-1.072)	0.70	1.021 (0.949-1.097)	0.581	1.009 (0.935-1.090)	0.815	1.430 (1.001-2.043)	0.049
**Chemotherapy cycle**		0.813		0.628		0.591		0.296		0.374		0.309
**Within 12 weeks**	reference		reference		reference		Reference		Reference		Reference	
**over 12 weeks**	1.030 (0.783-1.354)		0.928 (0.688-1.253)		0.921 (0.683-1.243)		0.838 (0.602-1.167)		0.860 (0.618-1.199)		0.842 (0.605-1.173)	
**Endocrine therapy**		0.813		0.822		0.859		0.751		0.513		0.527
**Not done**	reference		reference		reference		Reference		Reference		Reference	
**Done**	0.963 (0.703-1.318)		1.038 (0.749-1.440)		1.030 (0.743-1.428)		1.067 (0.713-1.597)		1.150 (0.756-1.750)		1.145 (0.753-1.740)	
**HER2-target therapy**		0.259		0.361		0.347		0.438		0.052		0.049
**Not done**	reference		reference		reference		Reference		Reference		Reference	
**Done**	1.180 (0.885-1.573)		1.149 (0.853-1.547)		1.153 (0.857-1.551)		1.152 (0.805-1.648)		1.426 (0.997-2.039)		1.430 (1.001-2.043)	
**Radiotherapy**		0.004		0.003		0.003		0.034		0.052		0.049
**Not done**	reference		reference		reference		Reference		Reference		Reference	
**Done**	1.504 (1.143-1.978)		1.580 (1.171-2.134)		1.582 (1.172-2.136)		1.453 (1.029-2.050)		1.426 (0.997-2.069)		1.430 (1.001-2.043)	
**Lymph edema**		<0.001		0.002		0.002		0.033		0.816		0.055
**No**	reference		reference		reference		reference		reference		reference	
**Yes**	1.907 (1.336-2.722)		1.799 (1.235-2.619)		1.821 (1.252-2.649)		1.671 (1.042-2.682)		1.558 (0.946-2.566)		1.625 (0.989-2.669)	
**Axillary surgery**		0.141		0.031		0.033		0.012		0.009		0.012
**SLNB**	reference		reference		reference		reference		reference		reference	
**ALND**	0.809 (0.610-1.073)		0.718 (0.531-0.970)		0.721 (0.534-0.974)		0.649 (0.464-0.910)		0.627 (0.442-0.890)		0.640 (0.451-0.907)	
**Diabetes**		0.192		0.526		0.544		0.566		0.923		0.873
**No**	reference		reference		reference		reference		reference		reference	
**Yes**	1.392 (0.847-2.286)		1.190 (0.695-2.036)		1.181 (0.690-2.020)		1.219 (0.620-2.394)		1.036 (0.505-2.128)		1.061 (0.517-2.178)	

**Dyslipidemia**		0.397		0.814		0.816		0.542		0.829		0.899
**No**	reference		reference		Reference		reference		reference		reference	
**Yes**	1.142 (0.840-1.552)		0.959 (0.690-1.336)		0.962 (0.692-1.336)		1.124 (0.772-1.634)		0.957 (0.641-1.428)		0.974 (0.652-1.456)	
**Autoimmune disease**		0.345				0.592		0.168				0.248
**No**	reference				reference		reference				reference	
**Yes**	1.212 (0.813-1.806)				1.118 (0.744-1.680)		1.394 (0.869-2.238)				1.334 (0.818-2.177)	
**Rheumatoid arthritis**		0.212		0.458				0.883		0.986		
**No**	reference		Reference				reference		reference			
**Yes**	1.472 (0.802-2.703)		1.268 (0.677-2.373)				1.069 (0.438-2.611)		0.991 (0.400-2.459)			
**Lupus erythematous**		0.384		0.483				0.546				
**No**	reference		reference				reference					
**Yes**	2.394 (0.335-17.087)		2.052 (0.275-15.313)				2.357 (0.145-38.255)					
**Systemic connective tissue disorder**		0.262						0.299				
**No**	reference						reference					
**Yes**	4.918 (0.304-79.530)						4.385 (0.270-71.153)					
**Sicca syndrome**		0.384						0.324				
**No**	Reference						reference					
**Yes**	4.248 (0.263-68.651)						4.063 (0.250-65.943)					
**Psoriasis**		0.290		0.311				0.338				
**No**	Reference		Reference				reference					
**Yes**	0.346 (0.049-2.464)		0.362 (0.051-2.586)				0.256 (0.016-4.157)					
**Behcet's disease**		0.308						0.324				
**No**	Reference						reference					
**Yes**	4.248 (0.263-68.651)						4.063 (0.250-65.943)					
**Autoimmune thyroiditis**		0.735		0.537				0.601		0.420		
**No**	Reference		Reference				reference		reference			
**Yes**	0.786 (0.195-3.164)		0.642 (0.157-2.618)				0.591 (0.083-4.229)		0.440 (0.060-3.241)			
**Atopic dermatitis**		0.186		0.297				0.001		0.002		
**No**	Reference		Reference				reference		reference			
**Yes**	1.426 (0.843-2.413)		1.327 (0.780-2.259)				2.437 (1.426-4.165)		2.411 (1.384-4.200)			

**Vitiligo**	0.860			0.896		
**No**	Reference			reference		
**Yes**	0.778 (0.048-12.573)			0.831 (0.051-13.482)		
**Steroid medication**	0.536	0.633	0.568	0.627	0.824	0.788
**No**	Reference	Reference	Reference	reference	reference	reference
**Yes**	1.187 (0.690-2.042)	1.143 (0.660-1.981)	1.173 (0.678-2.028)	1.182 (0.602-2.322)	1.081 (0.543-2.152)	1.099 (0.552-2.187)

CCI, Charlson Comorbidity index; HER2, human epidermal growth factor receptor 2, SLNB, sentinel lymph node biopsy; ALND, axillary lymph node dissection

*Multivariate analysis model 1: If any of the autoimmune diseases, such as rheumatoid arthritis, lupus erythematous or systemic sclerosis etc, are present, the variable is defined as comprehensive autoimmune disease

*Multivariate analysis model 2: Analysis including each autoimmune disease as variables

Table S8. Risk of developing implant contracture in breast cancer patients according to the duration of chemotherapy in the TEI cohort using the Cox proportional hazard model

	**Before matching**						**After matching**					
	**Univariate analysis**		**Multivariate analysis model 1***		**Multivariate analysis model 2****		**Univariate analysis**		**Multivariate analysis model 1***		**Multivariate analysis model 2****	
	**HR (95% CIs)**	***P* value**	**HR (95% CIs)**	***P* value**	**HR (95% CIs)**	***P* value**	**HR (95% CIs)**	***P* value**	**HR (95% CIs)**	***P* value**	**HR (95% CIs)**	***P* value**
**Age (per 10-year)**	1.219 (1.055-1.407)	0.007	1.217 (1.045-1.418)	0.012	1.215 (1.044-1.415)	0.012	1.187 (0.994-1.417)	0.058	1.212 (1.006-1.461)	0.044	1.195 (0.992-1.439)	0.061
**CCI (Weight number)**	1.041 (0.990-1.095)	0.118	1.018 (0.963-1.076)	0.526	1.560 (1.169-2.083)	0.473	1.017 (0.949-1.090)	0.625	1.009 (0.938-1.086)	0.804	1.519 (1.073-2.150)	0.018
**Chemotherapy cycle**		0.812		0.727		0.678		0.790		0.789		0.787
**Within 12 weeks**	Reference		Reference		Reference		Reference		Reference		Reference	
**over 12 weeks**	1.032 (0.793-1.344)		0.950 (0.713-1.267)		0.941 (0.706-1.254)		0.957 (0.690-1.325)		0.956 (0.690-1.326)		0.956 (0.689-1.325)	
**Endocrine therapy**		0.760		0.921		0.963		0.479		0.760		0.750
**Not done**	Reference		Reference		Reference		Reference		Reference		Reference	
**Done**	0.954 (0.706-1.290)		1.016 (0.744-1.388)		1.007 (0.737-1.376)		0.872 (0.596-1.275)		0.940 (0.634-1.395)		0.938 (0.632-1.392)	
**HER2-target therapy**		0.496		0.618		0.595		0.383		0.437		0.432
**Not done**	Reference		Reference		Reference		Reference		Reference		Reference	
**Done**	1.103 (0.832-1.460)		1.077 (0.805-1.439)		1.082 (0.810-1.445)		1.171 (0.822-1.668)		1.156 (0.802-1.666)		1.157 (0.804-1.666)	
**Radiotherapy**		0.003		0.003		0.003		0.014		0.021		0.018
**Not done**	Reference		Reference		Reference		Reference		Reference		Reference	
**Done**	1.491 (1.144-1.943)		1.557 (1.166-2.079)		1.560 (1.169-2.083)		1.527 (1.089-2.140)		1.507 (1.064-2.134)		1.519 (1.073-2.150)	
**Lymph edema**		<0.001		0.002		0.001		0.053		0.123		0.095
**No**	Reference		Reference		Reference		Reference		Reference		Reference	
**Yes**	1.887 (1.336-2.667)		1.797 (1.248-2.589)		1.824 (1.267-2.624)		1.609 (0.993-2.606)		1.490 (0.897-2.474)		1.535 (0.928-2.540)	
**Axillary surgery**		0.046		0.004		0.004		0.070		0.064		0.065
**SLNB**	Reference		Reference		Reference		Reference		Reference		Reference	
**ALND**	0.761 (0.581-0.996)		0.657 (0.493-0.874)		0.660 (0.496-0.878)		0.737 (0.529-1.026)		0.722 (0.512-1.019)		0.724 (0.514-1.020)	
**Diabetes**		0.123		0.452		0.463		0.971		0.668		0.689
**No**	Reference		Reference		Reference		Reference		Reference		Reference	

**Yes**	1.447 (0.904-2.316)		1.216 (0.731-2.022)		1.210 (0.727-2.012)		0.987 (0.484-2.014)		0.848 (0.399-1.802)		0.857 (0.402-1.826)	
**Dyslipidemia**		0.405		0.452		0.786		0.902		0.431		0.482
**No**	Reference		Reference		Reference		Reference		Reference		Reference	
**Yes**	1.134 (0.843-1.525)		1.216 (0.731-2.022)		0.957 (0.696-1.315)		0.976 (0.662-1.439)		0.847 (0.561-1.280)		0.863 (0.572-1.302)	
**Autoimmune disease**		0.343				0.615		0.087				0.149
**No**	Reference				Reference		Reference				Reference	
**Yes**	1.202 (0.822-1.757)				1.105 (0.749-1.629)		1.487 (0.944-2.341)				1.411 (0.884-2.253)	
**Rheumatoid arthritis**		0.201		0.485				0.424		0.670		
**No**	Reference		Reference				Reference		Reference			
**Yes**	1.462 (0.817-2.617)		1.239 (0.679-2.259)				1.363 (0.638-2.911)		1.186 (0.542-2.595)			
**Lupus erythematous**		0.483		0.572				0.424				
**No**	Reference		Reference				Reference					
**Yes**	2.021 (0.284-14.405)		1.784 (0.240-13.230)				1.922 (0.118-31.212)					
**Systemic connective tissue disorder**		0.275						0.291				
**No**	Reference						Reference					
**Yes**	4.717 (0.292-76.216)						4.488 (0.277-72.767)					
**Sicca syndrome**		0.320						0.312				
**No**	Reference						Reference					
**Yes**	4.103 (0.254-66.270)						4.207 (0.259-68.263)					
**Psoriasis**		0.251		0.269				0.361				
**No**	Reference		Reference				Reference					
**Yes**	0.317 (0.045-2.255)		0.331 (0.046-2.356)				0.273 (0.017-4.421)					
**Behcet's disease**		0.320						0.312				
**No**	Reference						Reference					
**Yes**	4.103 (0.254-66.270)						4.207 (0.259-68.263)					
**Autoimmune thyroiditis**		0.582		0.417				0.757		0.793		
**No**	Reference		Reference				Reference		Reference			
**Yes**	0.677 (0.168-2.720)		0.559 (0.138-2.273)				1.246 (0.309-5.034)		0.824 (0.194-3.505)			
**Atopic dermatitis**		0.149		0.232				0.009		0.013		

**No**	Reference		Reference				Reference		Reference			
**Yes**	1.440 (0.878-2.363)		1.357 (0.823-2.237)				2.097 (1.208-3.639)		2.053 (1.163-3.624)			
**Vitiligo**		0.837						0.991				
**No**	Reference						Reference					
**Yes**	0.746 (0.046-12.041)						1.016 (0.063-16.474)					
**Steroid medication**		0.666		0.757		0.703		0.816		0.931		0.942
**No**	Reference		Reference		Reference		Reference		Reference		Reference	
**Yes**	1.127 (0.656-1.936)		1.090 (0.631-1.884)		1.112 (0.644-1.919)		1.088 (0.534-2.220)		1.032 (0.502-2.124)		1.027 (0.498-2.119)	

CCI, Charlson Comorbidity index; HER2, human epidermal growth factor receptor 2, SLNB, sentinel lymph node biopsy; ALND, axillary lymph node dissection

*Multivariate analysis model 1: If any of the autoimmune diseases, such as rheumatoid arthritis, lupus erythematous or systemic sclerosis etc, are present, the variable is defined as comprehensive autoimmune disease

*Multivariate analysis model 2: Analysis including each autoimmune disease as variables

Figure S1. Schematic diagram illustrating the patient's enrollment and exclusion criteria

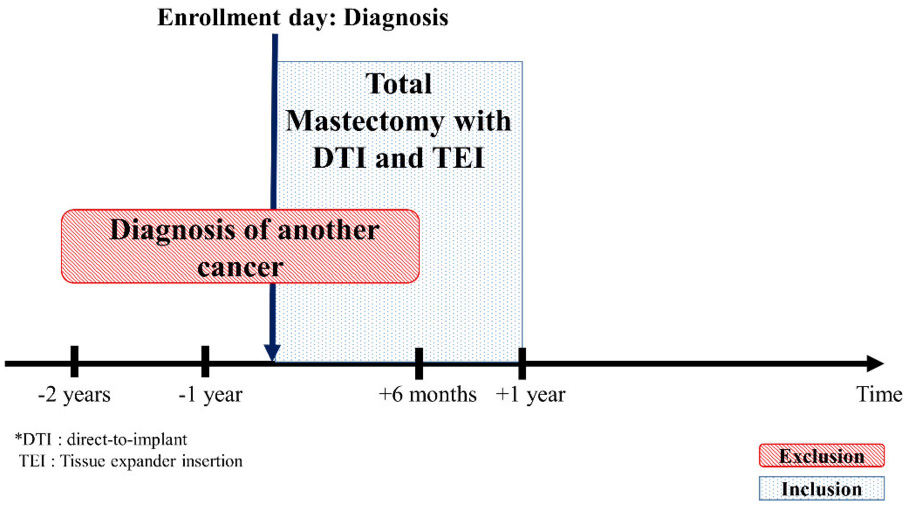

Data were obtained from the NHI database (2015–2018) for patients diagnosed with DCIS or breast cancer. Patients who did not undergo curative surgery within 1 year of diagnosis were excluded. The final analysis included only those who underwent total mastectomy with either DTI or TEI reconstruction and received chemotherapy

Figure S2. Cumulative incidence of capsular contracture in breast cancer patients undergoing DTI surgery by chemotherapy duration

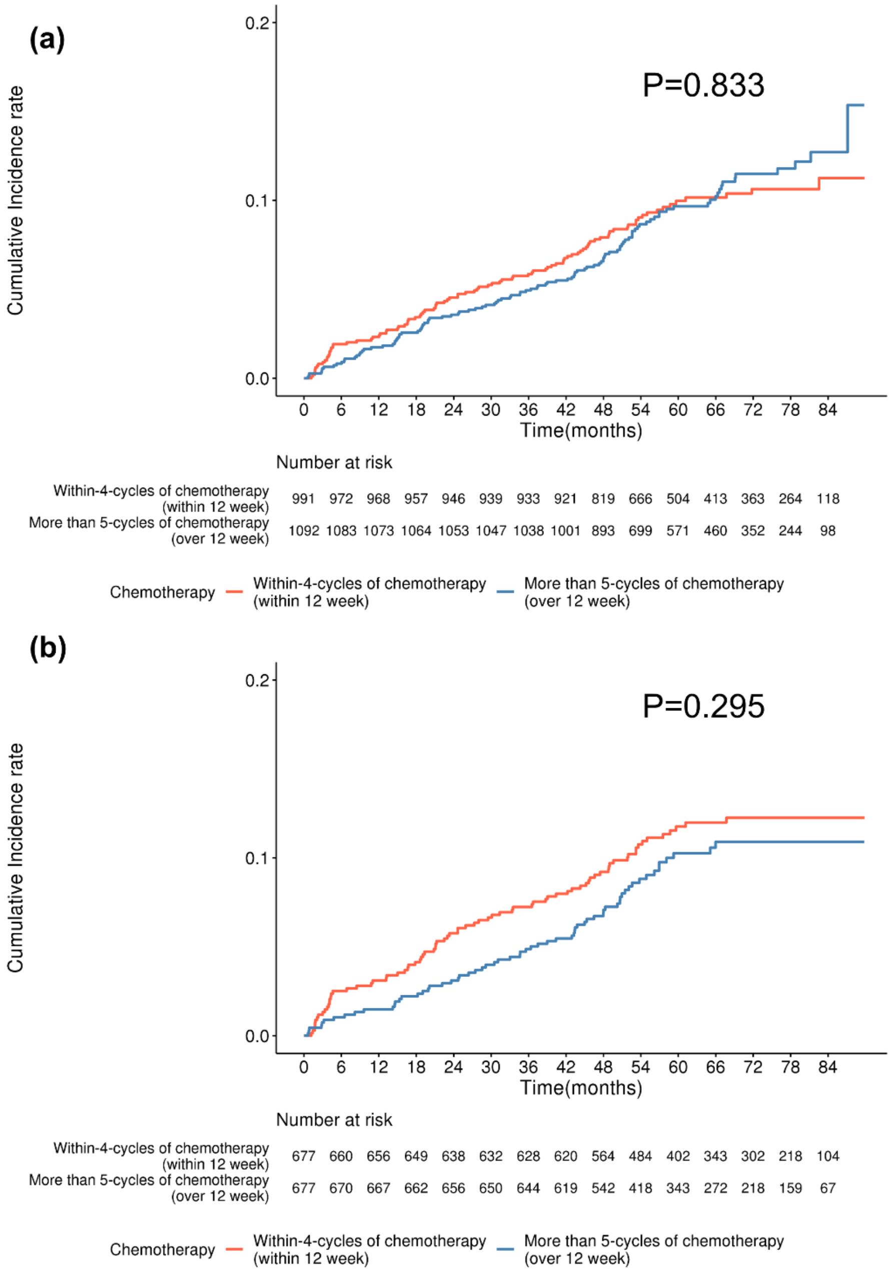

(a) Before matching, no significant difference was observed in the cumulative incidence of capsular contracture based on chemotherapy duration (p = 0.833, log-rank test). (b) After matching, the difference remained statistically nonsignificant (p = 0.295, log-rank test).

Figure S3. Cumulative incidence of capsular contracture in breast cancer patients undergoing TEI surgery by chemotherapy duration

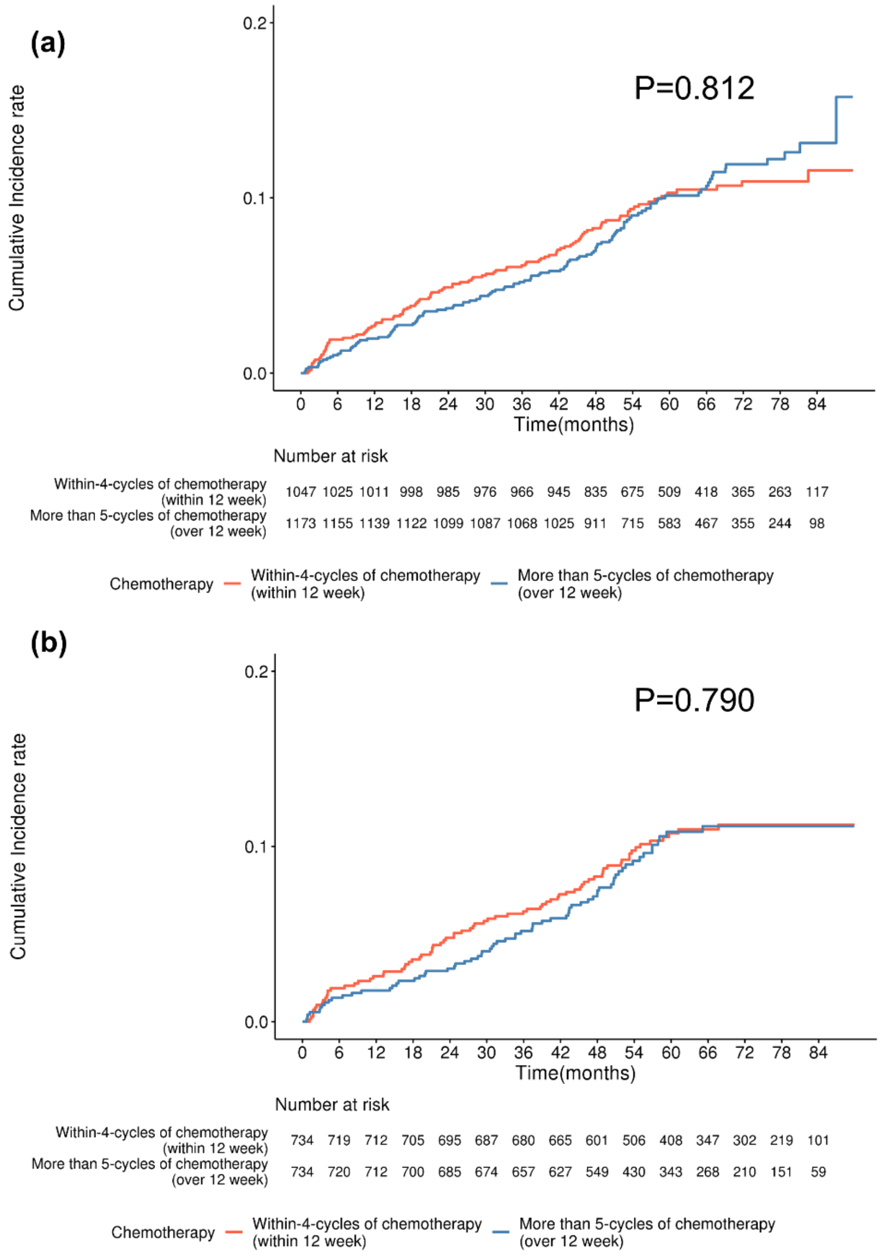

(a) Before matching, there was no significant difference in the cumulative incidence of capsular contracture based on chemotherapy duration (p = 0.812, log-rank test). (b) After matching, the difference remained statistically nonsignificant (p = 0.790, log-rank test)
